# Seasonal changes in network connectivity and consequences for pathogen transmission in a solitary carnivore

**DOI:** 10.1038/s41598-023-44815-y

**Published:** 2023-10-18

**Authors:** Marie L. J. Gilbertson, S. Niamh Hart, Kimberly VanderWaal, Dave Onorato, Mark Cunningham, Sue VandeWoude, Meggan E. Craft

**Affiliations:** 1 Department of Veterinary Population Medicine, University of Minnesota, St Paul, MN 55108 USA; 2 Fish and Wildlife Research Institute, Florida Fish and Wildlife Conservation Commission, Naples, FL 34114 USA; 3 Fish and Wildlife Research Institute, Florida Fish and Wildlife Conservation Commission, Gainesville, FL 32601 USA; 4 Department of Microbiology, Immunology, and Pathology, Colorado State University, Fort Collins, CO 80523 USA; 5 Department of Ecology, Evolution and Behavior, University of Minnesota, St Paul, MN 55108 USA; 6 Present Address: Wisconsin Cooperative Wildlife Research Unit, Department of Forest and Wildlife Ecology, University of Wisconsin–Madison, Madison, WI 53706 USA

**Keywords:** Ecology, Behavioural ecology, Conservation biology, Ecological epidemiology

## Abstract

Seasonal variation in habitat use and animal behavior can alter host contact patterns with potential consequences for pathogen transmission dynamics. The endangered Florida panther (*Puma concolor coryi*) has experienced significant pathogen-induced mortality and continues to be at risk of future epidemics. Prior research has found increased panther movement in Florida’s dry versus wet seasons, which may affect panther population connectivity and seasonally increase potential pathogen transmission. Our objective was to determine if Florida panthers are more spatially connected in dry seasons relative to wet seasons, and test if identified connectivity differences resulted in divergent predicted epidemic dynamics. We leveraged extensive panther telemetry data to construct seasonal panther home range overlap networks over an 11 year period. We tested for differences in network connectivity, and used observed network characteristics to simulate transmission of a broad range of pathogens through dry and wet season networks. We found that panthers were more spatially connected in dry seasons than wet seasons. Further, these differences resulted in a trend toward larger and longer pathogen outbreaks when epidemics were initiated in the dry season. Our results demonstrate that seasonal variation in behavioral patterns—even among largely solitary species—can have substantial impacts on epidemic dynamics.

## Introduction

Outbreaks of infectious diseases pose significant threats to the population health and conservation of free-ranging wildlife^1,2^, and seasonality can have profound impacts on the dynamics of these outbreaks^3^. Langwig et al.^4^ outlined five mechanisms through which seasonality may alter transmission dynamics via: (1) variation in sociality, (2) birth pulses causing influxes of new susceptibles, (3) variation in habitat use, (4) variation in climatic factors, and (5) variation in host immune function. Yet these are not necessarily mutually exclusive mechanisms. For example, climatic variation itself can drive changes in habitat use^5,6^ or animal social behaviors^7^. Given this covariation, an emergent question is whether seasonality contributes to significant changes in behavior and host contact patterns, which then translate to consequent changes in transmission dynamics.

While seasonal changes to host contact structure have been associated with predicted changes in outbreak size and speed of pathogen spread^8^, the relative impact of seasonality on pathogen dynamics likely varies by factors such as the magnitude of seasonality^9^, individual pathogen characteristics (e.g., incubation period), and local context. For example, seasonal variation in habitat selection by zebra (*Equus quagga*) in Namibia has been associated with increased anthrax mortalities in the wet season. However, during a period of severe drought, zebra altered their habitat selection with a consequent reduction in wet season anthrax mortalities^6^. The relative importance of seasonality on pathogen transmission may be of particular significance for species of conservation concern, for heavily managed populations, or in resource-limited conditions, where such seasonal effects may dictate optimal timing of surveillance, interventions, or other management activities to protect at-risk populations^10,11^.

One such relevant example is the Florida panther (*Puma concolor coryi*), an endangered subspecies of puma found only in south Florida. In the early 1990s, only an estimated 20–25 adult panthers remained^12^. Panther genetic diversity was extremely limited, suggestive of depressive inbreeding^12^, and appeared to be associated with increased disease burden among panthers^13,14^. Panthers have been infected with pathogens such as pseudorabies, rabies, notoedric mange, dermatophytosis, and feline leukemia virus (FeLV), which have all resulted in mortality^15^. Additionally, infectious agents including feline calicivirus, feline immunodeficiency virus, feline panleukopenia virus, and *Toxoplasma gondii* have all been documented in panthers^13,16,17^. FeLV spillover from domestic cats, in particular, was responsible for a deadly outbreak among panthers in 2002–2004^18,19^ and is an ongoing threat to the population^20,21^. While recent research has devoted much effort to discerning drivers of transmission among panthers (e.g.^22,23^), far less effort has been devoted to understanding how transmission dynamics among panthers may be subject to seasonal variations.

Southern Florida is subject to seasonal climatic variability with a tropical monsoon climate, wherein 75% of total annual precipitation falls on the landscape during the wet season (from May to October^24^). Ground that is dry one month can shift to non-traversable swampland the next. Such hydrologic changes have been implicated in observed seasonal variation in movement patterns among Florida panthers, with increased movement observed in the dry season^25,26^. Although contact rates between puma (*Puma concolor*) in the Greater Yellowstone area have been shown to vary seasonally^27^, it is unknown if seasonal hydrological changes in the subtropical climate of south Florida and their effect on panther movement or behavior translate to changes in panther population connectivity. Further, it is unclear if seasonal changes in population connectivity would impart measurable, biologically significant impacts on epidemic outcomes.

The objectives of this study were to determine (1) if the panther population is more spatially connected in dry seasons than wet seasons, as measured by home range overlap, and (2) if any differences in connectivity could result in different epidemic outcomes in dry versus wet seasons, and identify transmission conditions under which those difference may be the greatest. We use the term *spatial connectivity,* hereafter, to refer to the connectedness of individuals within our population as a result of home range or spatial overlap. Results of this study shed light on the impact of social ecology on subsequent pathogen transmission and management in free-ranging wildlife.

## Materials and methods

We used very high frequency (VHF) telemetry data previously collected from Florida panthers captured and radiocollared by the Florida Fish and Wildlife Conservation Commission and National Park Service. Captures followed FWC agency guidelines for the immobilization and handling of wild panthers, which have been modeled to closely follow the American Society of Mammalogists’ guidelines for the use of wild mammals in research^28^. We selected the years of 1996–2007 as our study period, as telemetry coverage during this window showed consistent, high numbers of monitored individuals, with a mean of 36 panthers monitored per year (range 26 to 47; minimum panther population size was approximately 30–90 individuals during this period^29^). Relocations of radiocollared panthers were recorded from aircraft during the day, typically three times per week. We followed Criffield et al*.*^25^ in defining the south Florida wet season as 15 May through 14 October, and dry season as 15 October through 14 May each year (Fig. S1). We reviewed telemetry locations for each individual in each season per year to identify errant or outlier points that were likely erroneous based on extreme divergence from all other points, implying unrealistic speeds of travel; this process resulted in the removal of 13 relocations across the 11 year dataset.

### Panther seasonal spatial connectivity

We evaluated seasonal changes in the potential for individuals to interact by estimating spatial overlap between panther home ranges in each year and season^30–32^. We chose spatial overlap as a representation of potential interactions because our data were limited to relocations collected three times per week, which precluded reliably identifying direct contact events. Spatial overlap is representative of the potential for individuals to interact indirectly, and can be representative of direct interactions^30^, with this approach having been used previously in solitary (*Puma concolor*^33^) and social carnivores (wolves, *Canis lupus*, and lions, *Panthera leo*^34^). Further, the long-term nature of our relocation data provided a unique opportunity to examine seasonal patterns across an 11-year period; this is often not feasible with higher temporal resolution GPS collar data due to the shorter battery life of these devices^35^.

To quantify spatial overlap between panthers, we used the utilization distribution overlap index (UDOI^36^) with the 95% bivariate normal kernel home range. We chose to use UDOI, as it is the preferred metric when measuring space-use sharing between individuals (as compared to metrics such as volume of intersection and Bhattacharyya’s affinity)^36,37^. Home range kernels, 95% isopleth areas, and UDOI were estimated using the adehabitatHR package in R (R version 3.6.3 and 4.2.0^38,39^), as the coarseness of our relocation data did not require the use of autocorrelated kernel density estimators^40,41^.

Weighted networks were then constructed for each season and year, whereby individuals were connected in the network with edges weighted by UDOI (i.e., panthers with very little home range overlap had low edge weights). Our primary analysis did not subset networks by a minimum or threshold UDOI value (hereafter, *filter*), as such filtering is generally not recommended in network analysis^42^. However, we also replicated analyses with networks where edges were filtered for UDOI greater than or equal to 0.01 and 0.1 (an additional 44 networks) in order to test the sensitivity of our results to edge definitions.

For each network, we calculated node metrics of degree and strength, and network metrics of density and modularity^43^, all of which are key indicators of network connectivity and pathogen transmission^44^. *Degree* and *strength* are the number of connections and sum of weighted connections for each node, respectively. Individuals with high degree and/or strength may be candidates as “superspreaders”^45^. *Density* is a network-level measure of overall connectivity, while *modularity* is a measure of network subdivisions which may affect transmission dynamics^46^. Because networks were different sizes across seasons and years, we calculated the *normalized degree* for each node (degree divided by n-1, where n is the number of nodes in the network) to facilitate comparisons of networks across the study period. Strength was calculated using UDOI as the edge weight. Density was calculated as the proportion of realized versus possible edges in a network^47^. Modularity was calculated using a walktrap community-finding algorithm with 4 and 7 steps (to test sensitivity to this choice), with inverse UDOI as the edge weight. All network metrics were calculated using the *igraph* package in R^48^.

We tested for differences in the node- and network-level metrics in dry versus wet seasons. Because panthers were often monitored for multiple numbers of seasons and years (i.e., repeated measures within individuals), for node-level metrics (normalized degree and strength), we used a cluster level bootstrap^49^ to model metric values as a function of log home range area (in square kilometers) and season. We treated individual panthers as clusters (i.e., panthers were the unit that we resampled). Duration of observation (i.e., the number of seasons) was variable across the population, however, resulting in unequal cluster sizes, so our bootstrap approach sampled with replacement based on cluster size (we sampled with replacement individuals who were monitored for two seasons, three seasons, and so on)^49^. In each bootstrap iteration, we fitted the above linear model and recorded coefficient estimates (bootstrap statistics). We used 1000 iterations per bootstrap analysis, then extracted 95% confidence intervals for each coefficient from the quantiles of the resulting coefficient bootstrap distributions.

In addition, to specifically test if seasonal differences in panther connectivity were linked to hydrological changes (versus an unmeasured metric of seasonality), we compared our node-level metrics to seasonal precipitation. Precipitation data was accessed from the National Oceanic and Atmospheric Administration’s Climate Data Online service^50^. We averaged daily precipitation totals across Collier County weather stations from May 15, 1996 to May 14, 2007 and summed these averages per season per year to give seasonal *total average precipitation* values for each year and season. We used Spearman correlation to test for correlations between these total average precipitation values and median node-level metrics per season and year.

For the network-level metrics of density and modularity, we used non-parametric Kruskal–Wallis rank sum tests to test for differences in these metrics in the dry versus wet seasons across the 22 networks (2 seasons for each of 11 years). All Kruskal–Wallis tests were performed using the *stats* package in R^38^.

### Seasonal network connectivity and epidemic outcomes

We used a simulation approach to determine if differences in network connectivity alter epidemic outcomes in dry versus wet seasons. A simulation approach allowed us to examine transmission of a range of potential pathogens through empirically-informed networks, a strategy that can be helpful for highlighting the conditions or parameter space under which we expect to see the greatest impact of host or pathogen characteristics on epidemic outcomes^5,46,51–54^. Furthermore, network size was variable across years and seasons, and the degree distribution of networks can be influenced by network size and density^42^. Hence, a simulation approach allowed us to limit epistemic uncertainty (variation resulting from experimental uncertainty^55^) resulting from variation in network size, such that our simulation results would be indicative of differences between wet and dry season networks.

We simulated networks based on normalized degree and UDOI distributions from the observed networks, using separate distributions for dry and wet seasons. For each observed network, we fit a beta distribution to the normalized degree, and a gamma distribution to the observed UDOI edge weights using the *fitdistrplus* package in R^56^. We then took the mean of the beta and gamma distribution parameters across the dry and wet seasons, respectively. In addition, we recorded the number of isolates (completely unconnected individuals) in each observed network and, across all dry and all wet season networks, fit separate Poisson distributions to their respective counts of isolates. This approach resulted in three distributions per season describing normalized degree, UDOI, and the number of network isolates.

We then simulated new networks based on these three distributions for each season (degree, UDOI edge weight, and number of isolates). Simulated networks each had 33 nodes, which was the average network size across all observed networks. We simulated networks as single large components (rather than two or more subcomponents), then added isolated individuals by drawing from the corresponding dry or wet season Poisson distribution. Among non-isolates, we simulated a normalized degree distribution from the corresponding dry or wet season beta distribution, and transformed these values to standard degree values based on the population size. We then used simulated annealing in the *ergm* package in R^57^ to generate a network with the target degree distribution. Simulated annealing is not always precise, and because we did not detect statistically significant differences in network density between dry and wet seasons (see results), we constrained simulated network density to ± 25% of the average network density across all networks.

To assign edge weights, we followed Reynolds et al*.*^8^ and randomly assigned edge weights by drawing from the corresponding gamma distribution for dry versus wet season UDOI values. There was no evidence of correlation between degree and UDOI (treating each UDOI estimate as independent, Kendall’s τ =  − 0.06), supporting random assignment of UDOI values in our simulations. Because UDOI values can be greater than one^36^, we scaled simulated UDOIs by the maximum drawn UDOI per simulated network so all values fell between 0 and 1 for transmission simulations (see Fig. S2 for representative examples of simulated networks).

Network metrics that reflect higher order or indirect network structure (e.g., clustering coefficient, betweenness) are typically less robust to undersampling of populations^58–60^, so we did not simulate networks constrained by these metrics. The impact of undersampling on modularity is less clear^60^, and we did not specifically constrain the modularity of simulated networks. However, to ensure our simulated epidemic outcomes were robust to higher order network structure (in the form of modularity), we evaluated epidemic outcomes (see below) for the subset of simulations in which simulated network modularity was within the bounds of observed modularity in respective dry and wet seasons.

With each simulated network, we simulated pathogen transmission through that network in weekly time steps. Transmission was initiated with a randomly selected individual and simulations continued for a maximum of six months (representing the length of our seasons). Because our networks were based on spatial overlap between panthers, we expected that our simulations would be most representative of indirectly or environmentally transmitted pathogens (e.g., gastrointestinal helminths, feline panleukopenia virus). However, spatial overlap has been used to represent the potential for direct social interactions between *Puma concolor* and bobcat (*Lynx rufus*)^33^, as well as direct intraspecific associations in other species (e.g.^30–32^). Our simulations may therefore also have relevance to directly transmitted pathogens, assuming a direct relationship between spatial overlap and direct contact. As such, transmission simulations were designed to represent a broad range of potential pathogen types, including those that cause chronic infections (susceptible-infectious model, SI; e.g. directly transmitted feline immunodeficiency virus), those that cause infections that can recur after recovery (susceptible-infectious-susceptible model, SIS; e.g. many indirectly transmitted gastrointestinal helminths), and those that cause infections that induce immunity (susceptible-infectious-recovered model, SIR; e.g. directly or indirectly transmitted feline panleukopenia virus).

Transitions between infection states occurred through a stochastic chain binomial process. In each time step, transmission (transition from susceptible to infectious) occurred for an individual if all the following conditions were met: (1) the existence of an edge between a susceptible and infectious individual; (2) a random binomial draw based on scaled UDOI edge weight (high edge weight = high probability of success); (3) a random binomial draw based on an additional edge weight scaling parameter, *ρ*, used to adjust the relative effect of UDOI-based edge weights; and (4) a random binomial draw based on the probability of transmission, given contact, represented by *β* (note that this is different from the conventional *β* in most compartmental models). UDOI can range from zero to greater than one, and even scaled to range from zero to one, empirical evidence from *Puma concolor* demonstrates that it is unlikely that even highly overlapping panthers interact on a weekly basis^27^. We therefore included the parameter *ρ* to scale UDOI-based contact probabilities to better represent the expected low rate of weekly contact in panthers. Further, inclusion of *ρ* allowed us to explicitly evaluate epidemic outcomes across a range of probabilities of contact. We repeated all simulations using binary (unweighted) edges instead of UDOI-weighted edges, but still including *ρ*. In SIS and SIR models, recovery from an infectious state (to susceptible or recovered, respectively) occurred at a rate γ, representing the weekly probability of recovery.

All parameters were varied across a range of parameter space in a full factorial design (Table 1). The combination of three model types (SI, SIS, SIR), weighted versus binary networks, and the potential values for the three transmission parameters (*ρ*, *β*, γ) resulted in 210 parameter sets which were instituted across dry and wet season simulation scenarios (for a total of 420 parameter sets). For each parameter set, a *full simulation* consisted of simulation of either a single dry or wet season network, and a transmission simulation through that network. 100 full simulations were completed for each parameter set in each of dry and wet season scenarios, resulting in a total of 42,000 full simulations.

**Table 1:** Transmission model types and simulation parameters.

Parameter	Parameter values
Model type	SI, SIS, SIR
Edge weight modifier (*ρ*)	0.6, 0.8, 1.0
Probability of transmission, given contact (*β*)	0.2, 0.4, 0.6, 0.8, 1.0
Weekly probability of recovery (*γ*)*	0.125, 0.25, 0.5

The parameter types and values used across the full factorial design for transmission simulations. Values represent probabilities used in random binomial draws. Each unique set of parameters was used for 100 simulations in each of dry and wet season scenarios.

*The weekly probability of recovery applied only to SIS and SIR model types.

To compare outcomes of simulated epidemics between dry and wet season scenarios, we recorded several key outcomes from each simulated epidemic. These included: outbreak duration (for SIS and SIR), total number of individuals ever infected, and proportion of outbreaks that failed. Failed outbreaks were defined as those that initiated in an unconnected isolate or in a non-isolate but only affected 1 individual; otherwise, epidemics were considered “successful”. We compared outcomes between wet and dry seasons using heat maps; for outbreak duration and total individuals infected, we compared mean values from successful epidemics. In addition, for the key result of “total ever infected”, we fit a generalized linear mixed model (GLMM), assuming a gamma distribution and log link. In this model, our response variable was the total number of panthers ever infected, with fixed effects for season and simulated network density, and a random intercept for parameter set (which incorporated model type). We controlled for simulated network density in this model to determine if differences in epidemic size were the result of season or simply differences in network density that emerged from simulations. Finally, to examine how our transmission parameters contributed to total infections and outbreak durations, we performed a variable importance analysis using the *randomForest* package in R^61,62^. For each of these two outcome variables, we performed a random forest regression with 2000 trees for all simulations infecting more than one individual. For outbreak duration, we only analyzed results from the SIR model type (these outbreaks were less likely to persist through the whole simulation duration).

## Results

### Panther spatial connectivity was higher in dry seasons than in wet seasons

**Figure 1:**
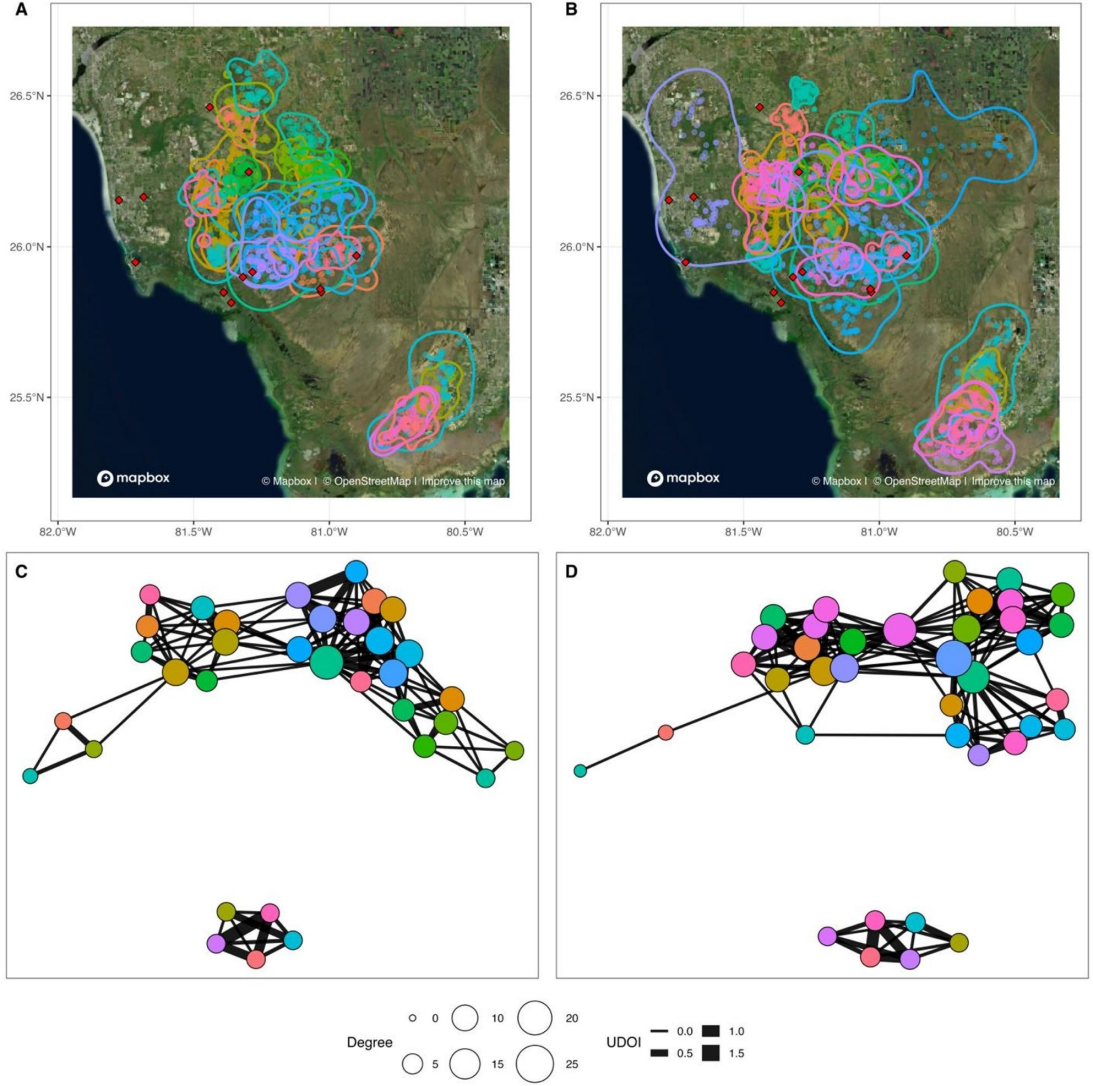
Florida panther 95% home range isopleths and locations (**A,B**) and home range overlap networks (**C,D**) from the wet (**A,C**) and dry (**B,D**) seasons in 2000. Locations of Collier county weather stations are indicated by red diamonds in (**A,B**). In (**C,D**), each node is a unique panther, node size reflects degree (number of connections in the network), and edge width corresponds to utilization distribution overlap index (UDOI). Node position in the networks does not correspond to geographic location. Colors of individuals match between the map and corresponding network (i.e., the teal individual in the central north of map B is the same teal individual at the left of the network in (**D**)), but do not match between maps or between networks. Results from 2000 were chosen as a representative example, with all networks available in Supplementary Figs. S3–S13. Maps were generated using Mapbox (https://www.mapbox.com/about/maps/) and OpenStreetMap (http://www.openstreetmap.org/copyright); Mapbox encourages map improvement feedback. (https://www.mapbox.com/map-feedback/).

**Figure 2:**
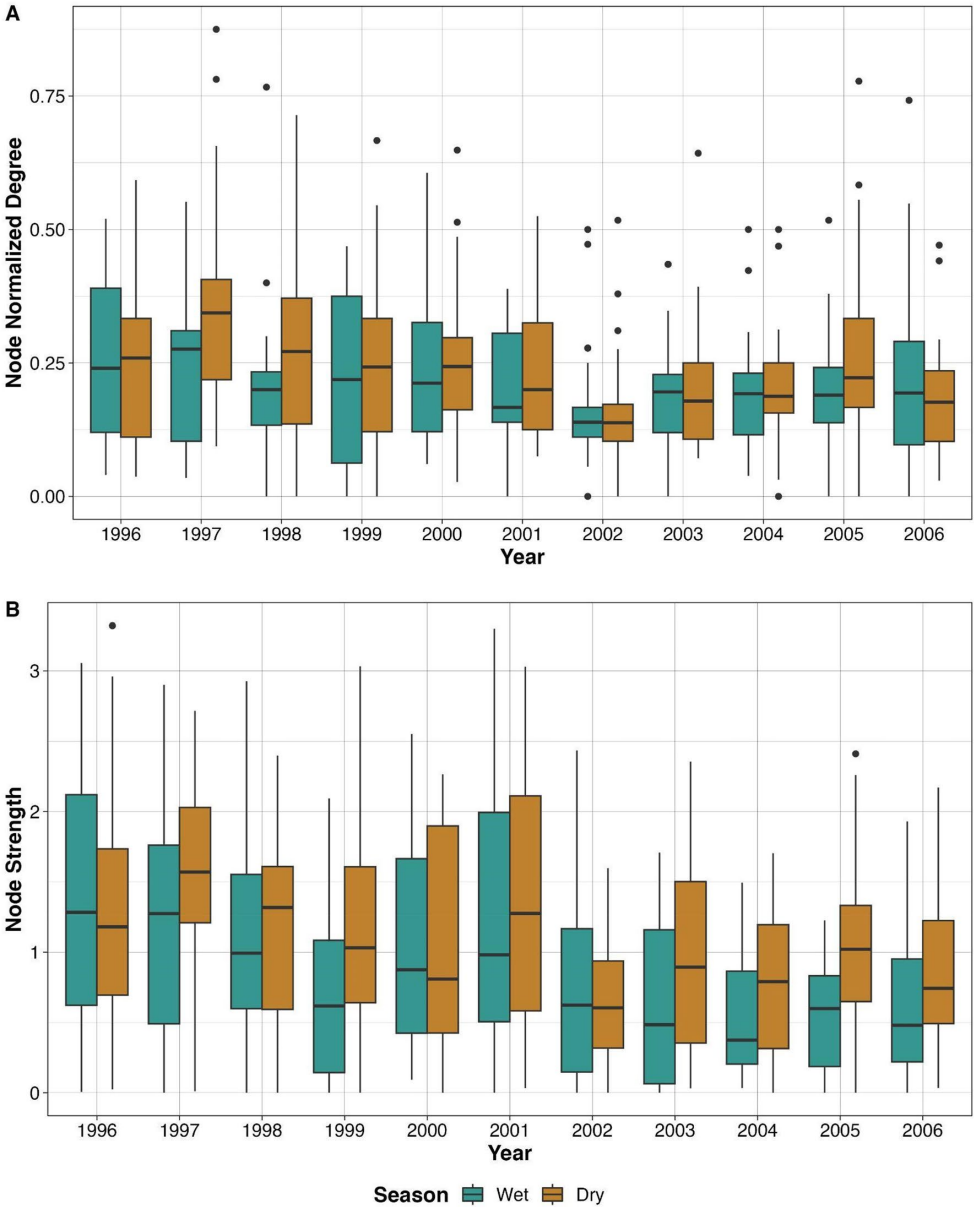
Node-level metrics from Florida panther spatial overlap networks in the context of season. (**A**) Shows normalized degree over time; (**B**) shows strength over time. In both panels, dry season results are dark tan and wet season results are aqua blue. All degree and strength results are from networks with no edge filtering.

**Figure 3:**
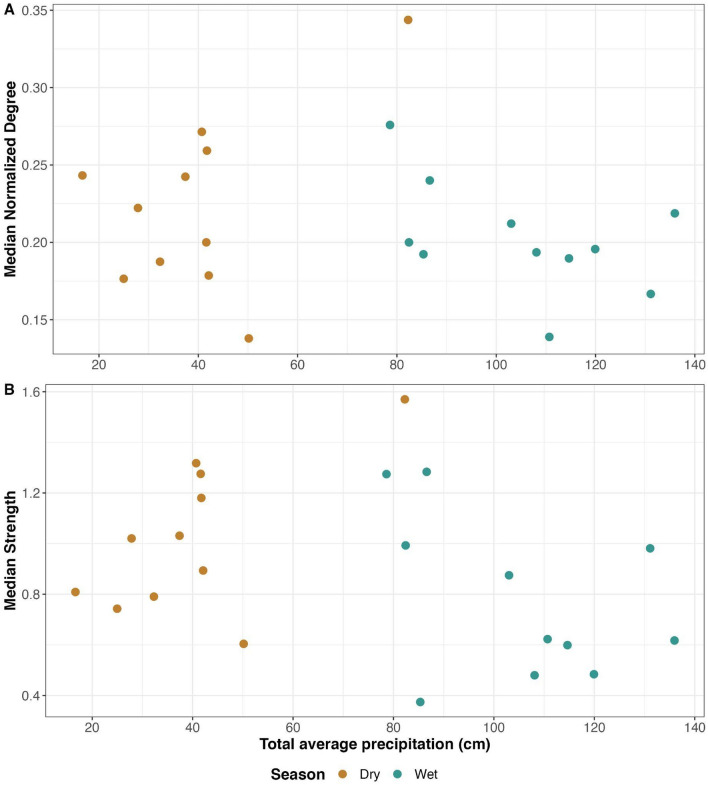
Among Florida panther spatial overlap networks, median values for node-level (**A**) normalized degree and (**B**) strength per season and year compared to seasonal precipitation. “Total average precipitation” was calculated per season and year, and averaged across Collier County weather stations. Dry seasons are colored dark tan, and wet seasons are in teal. All degree and strength results are from networks with no edge filtering.

Florida panther home range overlap networks were generally highly connected, with few observed unconnected individuals across the 22 seasonal networks (Fig. 1, Figs. S3–S13). Overlap, as quantified by UDOI, was right-skewed such that most pairs had low overlap and a smaller proportion had high degrees of overlap (Table S1, Fig. S14). Node-level metrics of normalized degree and strength were higher in dry seasons than in wet seasons (Fig. 2, Table 2). Because we used normalized degree as our response variable in the former model, effect sizes represent changes in node degree per n-1 individuals in a network. As such, seemingly small effect sizes can be associated with larger impacts in realized networks. Larger home range (HR) sizes contributed to higher node level metrics, but did not wholly account for the observed seasonal differences (Table 2, Figs. S15, S16). Furthermore, dry seasons had higher node-level metrics than wet seasons even when filtering edges by higher UDOI values (Table S2). Median values for both node-level metrics were positively correlated with seasonal total average precipitation in dry seasons, while wet seasons were negatively correlated, though these correlations did not achieve statistical significance (note small sample sizes; Fig. 3, Table S3).

**Table 2:** Cluster level bootstrap of node-level metrics from Florida panther spatial overlap networks.

Node metric	Variable	Estimate	Coefficient 95% CI
Normalized degree	Intercept	− 0.1	(− 0.2, 0.007)
	**Log(HR area)**	**0.06**	(0.04, 0.08)*
	**Season (Wet)**	− **0.02**	**(**− **0.03,** − 0.006)*
Strength	Intercept	0.04	(− 0.4, 0.5)
	**Log(HR area)**	**0.2**	(0.09, 0.3)*
	**Season (Wet)**	− **0.1**	**(**− **0.2,** − 0.08)*

Linear model coefficient estimates with 95% confidence intervals (CI) from cluster level bootstrap. Home range (HR) area was modeled as the log of the home range area in square km; dry season was the reference level for season, so coefficient estimates represent the effect of wet season on the respective node level metric.

95% CIs that do not include zero are considered statistically significant (bolded and marked by *), as they do not include the null hypothesis value^63^. Results are from networks with no edge filtering.

In contrast, network-level metrics showed little to no differences between wet and dry seasons, and this was largely consistent across levels of UDOI edge filtering (Table S4). Modularity was higher in dry seasons than wet seasons (median modularity with 4 steps: dry = 0.62, wet = 0.47; with 7 steps: dry = 0.64, wet = 0.47), but only without UDOI filtering for edges, and this difference did not reach statistical significance (with no edge filtering, rank sum = 3.75; *p* = 0.053; Table S4). Network density was not statistically significantly different between wet and dry seasons at any level of UDOI filtering (with no edge filtering, rank sum = 1.99; *p* = 0.16; Table S4).

### Greater dry season connectivity led to more infected individuals

**Figure 4:**
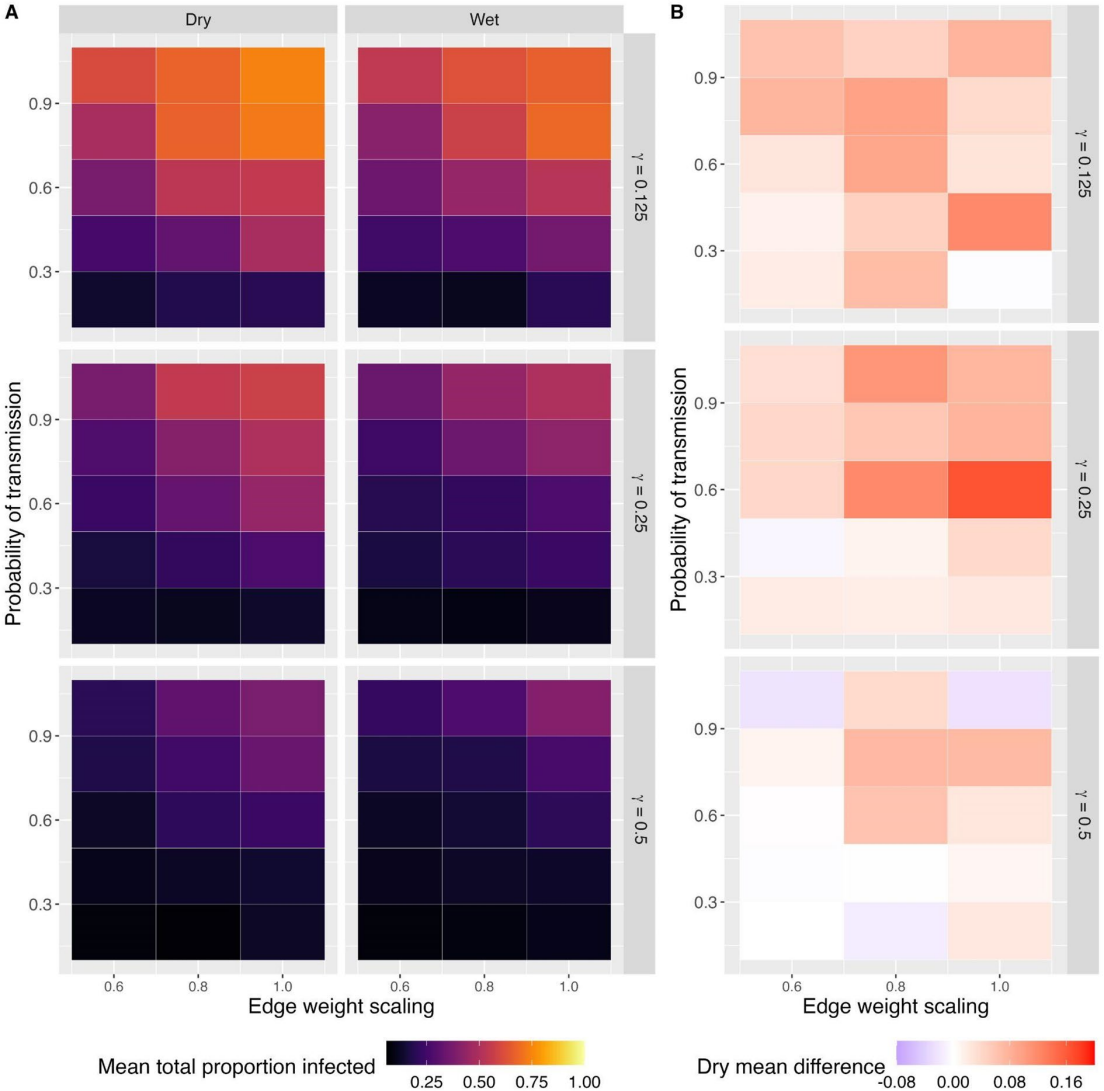
Heat maps from a SIR (susceptible-infectious-recovered) transmission model through Florida panther home range overlap networks showing (**A**) the mean total proportion of individuals infected in a simulated outbreak, and (**B**) the difference between these mean proportions for dry and wet seasons (relative to the dry season value) across simulation parameter space. The y-axes (left axes) give the probability of transmission. The panel rows (right axes) represent the weekly probability of recovery from infection (gamma), where a larger number equates to a faster recovery rate. In (**B**), red indicates more infections in the dry season, white is no difference, and purple represents more infections in the wet season.

Simulated epidemic outcomes demonstrated a trend toward more infected individuals, more successful epidemics, and longer durations of epidemics when the onset was in the dry season versus the wet season (Fig. 4, Figs. S17–S23). These findings were largely consistent across model types and parameter space. The maximum difference in mean proportion of individuals infected in the dry season relative to the wet season for SI, SIS, and SIR models was 12.5%, 17.8%, and 16.5%, respectively (Fig. 4, Figs. S17, S18). More generally, the greatest differences in number of individuals infected per season were typically apparent with intermediate to high transmission potential (combination of probability of transmission and edge weight scaling), and intermediate to long duration of infection (intermediate to low weekly probability of recovery; Fig. 4, Figs. S17, S18). When repeating simulations with networks with binary edges (no UDOI weighting), epidemics were typically much larger (often affecting all individuals) than in simulations with weighted edges, yet still showed a consistent trend for larger epidemics in the dry season than the wet season (Figs. S24–S26).

**Figure 5:**
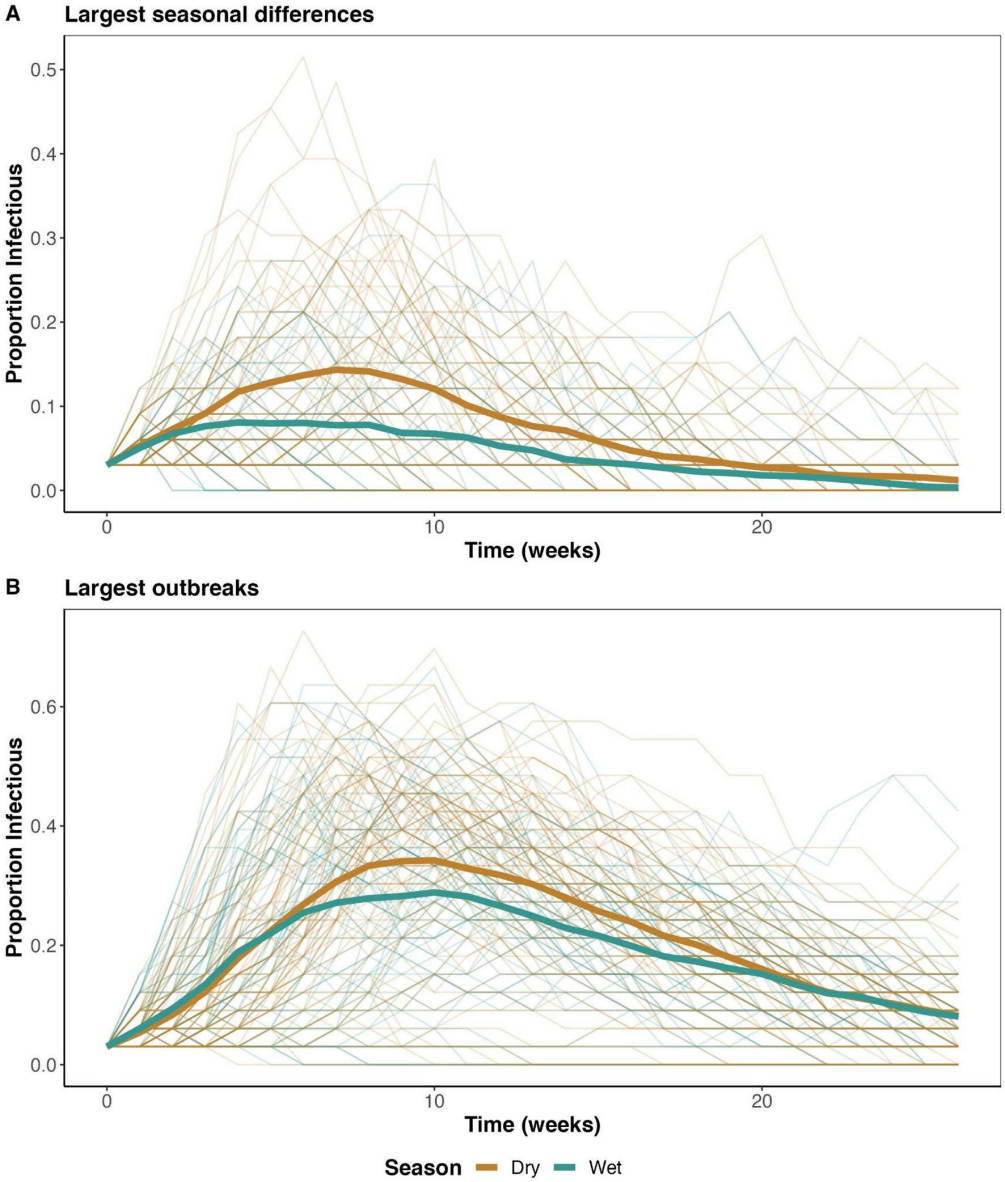
Epidemic curves from successful epidemics among simulated Florida panther populations with a SIR (susceptible-infectious-recovered) model. (**A**) Shows simulation results under the SIR parameterization conditions that produced the largest difference in total proportion of population infected between dry and wet seasons (probability of transmission given contact = 0.6, edge weight scaling = 1.0, weekly probability of recovery = 0.25). In contrast, (**B**) shows epidemic curves for the set of parameters producing the largest overall SIR epidemic sizes (probability of transmission given contact = 1.0, edge weight scaling = 1.0, weekly probability of recovery = 0.125). Dark tan lines show dry season results; teal lines show wet season results. Lighter lines are for individual simulations, and thick lines are mean values per time step across simulations. Note that y-axis scales differ between panels.

Examination of epidemic curves demonstrated that the differences in epidemic dynamics were more pronounced at some time points (Fig. 5, Fig. S27). For the SIR model producing the largest differences between dry and wet season outbreak sizes (probability of transmission given contact = 0.6, edge weight modifier = 1, weekly probability of recovery = 0.25), dry season epidemics had up to about two more infectious individuals at one time, compared to the wet season (Fig. S27). Because simulated population sizes were only 33 individuals, this reflects about 6% more of the population being infected at a single time point.

While observed networks did not show differences in density between dry and wet seasons, simulated network density, which was an emergent property of simulations, was higher in simulated dry season networks than wet season networks (Fig. S28). This was true despite having constrained densities to the same range of values for wet and dry seasons in our network simulation process, and therefore likely emerged from the differing underlying degree distributions. When accounting for simulated network density in our GLMM, season did not predict the total number of individuals infected in simulations (season: estimate = 0.01, standard error = 0.02, *p* = 0.7; network density: estimate = 5.4, standard error = 0.38, *p* < 0.001).

When evaluating the subset of simulation results in which simulated network modularity was within the ranges from observed networks, differences in epidemic sizes between dry and wet seasons were reduced but not eliminated (Figs. S29–S31). Again, this finding was true across model types and parameter space.

Our variable importance analysis found that the edge weight modifying and transmission probability parameters (*ρ* and *β*) were the most important for total simulated infections, and, along with the recovery probability parameter (γ), had a nonlinear relationship with this simulated outcome (Figs. S32, S33). In contrast, the recovery probability parameter (γ) was most important for the duration of SIR outbreaks, with a more linear relationship (Figs. S34, S35).

## Discussion

In this study, we first tested for differences in seasonal spatial connectivity in an endangered carnivore, the Florida panther. Our network approach indicated more and stronger spatial overlap among panthers during Florida’s dry season. We then determined if the observed differences in connectivity resulted in changes to predicted epidemic dynamics, finding that dry season outbreaks were consistently larger, of longer duration, and more likely to be successful.

We found that panther spatial overlap networks were more highly connected in dry seasons than wet seasons. This seasonality in overlap means that panthers likely have increased indirect contact in the dry season, and may also have increased direct contact during this period, assuming that direct contact is directly related to spatial overlap. Our results were consistent even when filtering network edges by higher home range overlap values (UDOI) and accounting for panther home range size, suggesting that the increased connectivity in dry seasons is not simply the result of weak home range overlap connections. Rather, seasonal behavioral changes or alterations in specific habitat use or movement likely contribute to increased spatial connectivity in panthers.

Panthers will reproduce throughout the year, but the majority of litters are born in February–June; with a 90 day gestation, this places most mating interactions in the dry season^64^. Furthermore, the dry season coincides with peak parturition of key panther prey species (i.e., white-tailed deer, *Odocoileus virginianus*, and feral swine, *Sus scrofa*^64^), which could alter panther habitat use as female panthers prioritize these young prey resources. Previous work has also implicated hydrological changes during the wet season with changes in panther movement; specifically decreased daily movements and shorter step lengths^25^. Decreased “ease of movement” during the wet season would explain the decreased spatial overlap connectivity we observed. In support of this hypothesis, we observed negative correlations between median values of node-level metrics of connectivity (degree and strength) and wet season precipitation (as total average precipitation), though this relationship did not achieve statistical significance. However, contrary to our expectations, we also observed positive correlations between node-level metrics and dry season precipitation, suggesting that spatial connectivity is the lowest in the driest dry seasons and the wettest wet seasons. This seemingly non-monotonic relationship between precipitation and spatial connectivity could be explained by increasing availability of usable habitat (e.g., unflooded) in the driest dry seasons, thereby reducing competition for spatially structured resources and consequent spatial overlap. In contrast, the wettest wet seasons may then favor increasing spatial isolation of individuals. Alternatively, if prey species become congregated in small areas during wet seasons, panthers may aggregate in these areas, facilitating increased competition and potential aggressive interactions. Aggregations of prey can also facilitate trophic transmission of pathogens like pseudorabies virus, where higher local prey densities increase intra-species transmission, thereby increasing the probability of spillover to predators^65^. The nature of interactions between panthers in areas of spatial overlap therefore merits investigation, particularly for the study of directly transmitted pathogens.

The mechanisms underlying the seasonal changes in spatial connectivity we observed here are likely nonlinear and multifactorial. Future work with higher resolution movement data could refine our understanding of the relationship between hydrology and panther space use, as well as examine if the changes we observed in panther spatial connectivity are due to increases in specific interactions (e.g., mating) or increased overlap in key habitat areas such as movement corridors or high-quality hunting areas. In panthers and other species of conservation concern, these key overlap areas may be particular regions to target for landscape preservation^66^ to conserve population genetic connectivity and key resources (e.g. hunting sites^67^). Alternatively, these could be locations supporting specific behaviors that can facilitate pathogen transmission^68,69^. For example, in territorial species, scent marking sites may be attractive during mating seasons but otherwise avoided, yielding seasonal differences in indirect contact and potential variation in pathogen outbreak dynamics^62,70^. Similarly, sharing kill sites of large prey may facilitate panther interactions (as in *Puma concolor*^71^) and consequent disease transmission as availability of larger prey varies over space or time. In addition, climate change is likely to compound or alter seasonal variation in spatial connectivity in panthers and other wildlife species. For example, sea level rise and precipitation changes as a result of climate change are expected to significantly alter Florida’s seasonal hydrological dynamics^72^. As such, future work should consider how such climate-induced landscape changes may affect resource availability, habitat quality and fragmentation, and how these changes may interact with other stressors to wildlife (e.g., human encroachment or alterations in prey availability) or additional climate change effects, such as increased survival of environmental pathogens^2^.

As a result of seasonal changes in panther spatial connectivity, simulated epidemics were consistently larger among panthers when an outbreak was initiated in the early dry season. This was particularly true for parameter space which may represent moderate to highly transmissible pathogens such as feline panleukopenia virus or feline herpesvirus^73^. Our variable importance analysis identified a potentially nonlinear relationship between outbreak size and the parameters representing pathogen transmissibility and scaled contact rates (*β* and *ρ*, respectively), with rapid increases in outbreak size observed at intermediate values of these parameters. This nonlinearity may contribute to a thresholding effect, above which outbreaks are more substantial and likely to produce biologically significant differences between seasons. While we lacked seasonal epidemiological data for specific pathogens in panthers, our results are consistent with observed seasonal differences in pathogen prevalence in other species, including cases that appear to be driven by seasonality in host contact patterns, behavior, or movement (e.g., rabies in several skunk species^74^; *Mycoplasma gallisepticum* in house finches, *Haemorhous mexicanus*^75,76^; phocine distemper virus in harbor seals, *Phoca vitulina*^77,78^). However, in the case of Tasmanian devils (*Sarcophilus harrisii*), observed seasonality in contact and biting patterns^79–81^ has not been associated with seasonal variation in new devil facial tumor disease cases^82^. This discrepancy may be the result of heterogeneity in the latent period before tumors become apparent^54^, and highlights that our results for simulated panther outbreaks could be altered by individual-level heterogeneities that we did not include here.

While observed dry season networks tended to be more modular—which might be expected to reduce outbreak size^44^—even when constraining simulation results to match this pattern, dry season outbreaks still resulted in more infected individuals than wet season outbreaks. This supports the conclusion that observed modularity was inadequate to overcome the increased transmission resulting from increased connectivity in dry seasons. Alternatively, our simulated networks were predominantly single large components, rather than multiple smaller subcomponents, the latter of which was observed in our empirical panther networks and might be expected to limit the extent of outbreaks. Panthers located in the Everglades at the far southeastern extent of panther habitat can be somewhat isolated from the broader population, but pathogens from the broader population can still reach these individuals^22^. Thus, our simulated, single component networks are still representative of broader population connectivity patterns, and reinforce the findings of Sah et al.^46^ that the ability of modularity to mitigate epidemics appears to be strongest with extremely high subdivision of networks.

The differences in predicted epidemic sizes between dry and wet seasons were typically minor but substantial in some cases, particularly for parameter space that generally produced intermediate to large epidemics (i.e., again most representative of pathogens such as feline panleukopenia virus or feline herpesvirus). When managing small populations, especially those such as panthers with limited host genetic diversity^12^, seemingly minor epidemic changes may pose significant risk to population health. For example, a cumulative increase in average infections approaching 18%—which we observed here—may represent substantial risk to population viability, where single individuals may be important for population genetic diversity^83^. Given that climate and land-use change are expected to increase novel inter-species viral transmission events^84^, we can expect that species of conservation concern like panthers will face new and emerging pathogen threats into the future. Our results help highlight seasonal vulnerability of the panther population and can direct management actions moving forward. For example, Florida panther capture and management activities predominantly occur during Florida’s dry season, and our results indicate this timing should be helpful for detecting emerging outbreaks when they could be more dangerous to population-level panther health.

The differences in epidemic outcomes that we observed here are likely mitigated by the low contact rates exhibited by *Puma concolor* more generally^27^. In contrast to solitary panthers, more gregarious or fission–fusion species would be expected to demonstrate larger differences in epidemic dynamics between seasons in the context of seasonal differences in connectivity. For example, seasonal contact differences in pastoral cattle herds in eastern Africa were found to result in significant changes in simulated epidemic dynamics^5^. The risks of sociality, however, may be mitigated by heterogeneity in individual associations^53^. Together, these results demonstrate the importance of linking movement, social, and disease ecology in order to understand the impact of seasonal space use and behavior changes on pathogen transmission.

An important observation in this study was finding that node-level metrics of connectivity were increased in dry seasons, while the network-level metric of connectivity, density, did not appear to vary between seasons. Prior research has shown that degree and strength are generally less sensitive to sampling limitations than density^60^. This suggests that our observed node-level metrics were more representative of connectivity differences than the observed densities. Interestingly, simulated network densities, emergent properties of our network simulations, did show differences between wet and dry seasons, and were an important predictor in simulated outbreak sizes. Degree distribution and density are closely linked^43^, suggesting that our empirical degree distributions should have been associated with more significant differences in network density than were captured by the sampling approach used here. As such, the predicted differences in outbreak dynamics by season likely represent a real but empirically unobserved effect of network connectivity, especially given the negligible effects of network modularity we observed. This finding highlights the importance of considering a suite of network attributes when assessing network connectivity and the effect of connectivity on outbreak dynamics.

This study made use of an extensive database of Florida panther relocations, but the frequency of relocations was limited to three weekly (which is a remarkable achievement for aerially relocated VHF collars over more than three decades). This limited our analysis of panther connectivity to an assessment of home range overlap. Home range overlap can sometimes be representative of direct contact and contact rates in free-ranging wildlife (e.g.^30^), and has been used previously to describe network structure in *Puma concolor*^33^. While research suggests that the amount of home range overlap may not correlate well with the number of direct contacts in territorial species, the spatial constraints imposed by hard habitat boundaries on the Florida panther population (along with a growing panther population^29,85^) result in a more aggregated population that is more amenable to extrapolating spatial overlap to direct contact network structures^60^. We therefore consider our simulations most relevant to indirectly transmitted pathogens, as well as potentially applicable to directly transmitted agents. Additional work comparing spatial overlap to direct contact rates in panthers and other wildlife species would help refine our understanding of when spatial overlap is most relevant as a proxy for direct contact rates. Thus, while we did not simulate specific pathogens and were limited to spatial overlap as an indicator of population connectivity, our results are able to set expectations for relative epidemic size between seasons across a range of pathogen types.

A significant uncertainty in our simulations was the expected weekly contact rate between panthers. We addressed this uncertainty by exploring epidemic dynamics across a range of possible contact rates, as represented by our edge weight scaling parameter; however, poorly characterized contact rates are an ongoing challenge in studies of panthers and other elusive wildlife species^35^. Further research with higher resolution movement data, especially in conjunction with proximity loggers, would be ideal to characterize contact rates and behaviors for future behavioral studies and pathogen transmission models. In addition, while we incorporated individual-level heterogeneity in contact patterns through our network approach, additional layers of heterogeneity are likely to affect seasonal variability in epidemic outcomes. For example, host immune response (i.e., susceptibility) or pathogen shedding may vary between individuals^86^ and seasons^3^, adding to the complexity of seasonal outbreak dynamics. Furthermore, while we assumed transmission within a single host species, multi-host pathogens are also affected by seasonality, including temporal variation in contact patterns between host species (e.g.^87^). As such, future work would benefit from examining how additional host and pathogen heterogeneities interact with seasonal dynamics to alter transmission processes.

## Conclusions

Florida panther spatial connectivity increased during south Florida’s dry season; these changes can result in substantial differences in epidemic dynamics, though these effects are likely mitigated by the low contact rates exhibited by this solitary carnivore. Regardless, ongoing conservation and management of panthers should benefit from a precedent of performing most capture and handling activities in the dry season, which may facilitate detection of outbreaks when they would have the greatest negative impact. This work demonstrates the importance of linking movement, social, and disease ecology to understand temporally dynamic risks to population health.

### Supplementary Information

Supplementary Information.

## Data Availability

Full R code for all analyses and simulations is available on GitHub (https://github.com/mjones029/Seasonal_panther_networks) and archived at Zenodo (10.5281/zenodo.10011452).

## References

[1] Breed AC, Delahay RJ, Smith GC, Hutchings MR (2009). Disease management in endangered mammals. Management of Disease in Wild Mammals.

[2] Smith KF, Acevedo-Whitehouse K, Pedersen AB (2009). The role of infectious diseases in biological conservation. Anim. Conserv..

[3] Altizer S (2006). Seasonality and the dynamics of infectious diseases. Ecol. Lett..

[4] Langwig KE (2015). Host and pathogen ecology drive the seasonal dynamics of a fungal disease, white-nose syndrome. Proc. Biol. Sci..

[5] VanderWaal K, Gilbertson M, Okanga S, Allan BF, Craft ME (2017). Seasonality and pathogen transmission in pastoral cattle contact networks. R. Soc. Open Sci..

[6] Huang Y-H (2021). Disease or drought: Environmental fluctuations release zebra from a potential pathogen-triggered ecological trap. Proc. Biol. Sci..

[7] Hirsch BT, Reynolds JJH, Gehrt SD, Craft ME (2016). Which mechanisms drive seasonal rabies outbreaks in raccoons? A test using dynamic social network models. J. Appl. Ecol..

[8] Reynolds JJH, Hirsch BT, Gehrt SD, Craft ME (2015). Raccoon contact networks predict seasonal susceptibility to rabies outbreaks and limitations of vaccination. J. Anim. Ecol..

[9] Donnelly R, Best A, White A, Boots M (2013). Seasonality selects for more acutely virulent parasites when virulence is density dependent. Proc. Biol. Sci..

[10] Baker L, Matthiopoulos J, Müller T, Freuling C, Hampson K (2019). Optimizing spatial and seasonal deployment of vaccination campaigns to eliminate wildlife rabies. Philos. Trans. R. Soc. Lond. B Biol. Sci..

[11] Oraby T, Vasilyeva O, Krewski D, Lutscher F (2014). Modeling seasonal behavior changes and disease transmission with application to chronic wasting disease. J. Theor. Biol..

[12] Johnson WE (2010). Genetic restoration of the Florida panther. Science.

[13] Roelke ME (1993). Seroprevalence of infectious disease agents in free-ranging Florida panthers (*Felis concolor coryi*). J. Wildl. Dis..

[14] Roelke ME, Martenson JS, O’Brien SJ (1993). The consequences of demographic reduction and genetic depletion in the endangered Florida panther. Curr. Biol..

[15] Onorato D, Macdonald DW, Loveridge AJ (2010). Long-term research on the Florida panther (*Puma concolor coryi*): Historical findings and future obstacles to population persistence. Biology and Conservation of Wild Felids.

[16] Carver S (2016). Pathogen exposure varies widely among sympatric populations of wild and domestic felids across the United States. Ecol. Appl..

[17] Gilbertson MLJ (2016). Is pathogen exposure spatially autocorrelated? Patterns of pathogens in puma (*Puma concolor*) and bobcat (*Lynx rufus*). Ecosphere.

[18] Brown MA (2008). Genetic characterization of feline leukemia virus from Florida panthers. Emerg. Infect. Dis..

[19] Cunningham MW (2008). Epizootiology and management of feline leukemia virus in the Florida puma. J. Wildl. Dis..

[20] Chiu ES (2019). Multiple introductions of domestic cat feline leukemia virus in endangered Florida panthers. Emerg. Infect. Dis..

[21] Gilbertson MLJ, Onorato D, Cunningham MW, VandeWoude S, Craft ME (2022). Paradoxes and synergies: Optimizing management of a deadly virus in an endangered carnivore. J. Appl. Ecol..

[22] Gilbertson MLJ (2022). Apathogenic proxies for transmission dynamics of a fatal virus. Front. Vet. Sci..

[23] Malmberg JL (2019). Altered lentiviral infection dynamics follow genetic rescue of the Florida panther. Proc. Biol. Sci..

[24] Abiy AZ, Melesse AM, Abtew W, Whitman D (2019). Rainfall trend and variability in Southeast Florida: Implications for freshwater availability in the Everglades. PLoS ONE.

[25] Criffield M (2018). Assessing impacts of intrinsic and extrinsic factors on Florida panther movements. J. Mammal..

[26] van de Kerk M (2015). Hidden semi-Markov models reveal multiphasic movement of the endangered Florida panther. J. Anim. Ecol..

[27] Elbroch LM, Quigley H (2016). Social interactions in a solitary carnivore. Curr. Zool..

[28] Sikes RS, Animal Care and Use Committee of the American Society of Mammalogists (2016). Guidelines of the American Society of Mammalogists for the use of wild mammals in research and education. J. Mammal..

[29] McBride RT, McBride RT, McBride RM, McBride CE (2008). Counting pumas by categorizing physical evidence. Southeast. Nat..

[30] Robert K, Garant D, Pelletier F (2012). Keep in touch: Does spatial overlap correlate with contact rate frequency?. J. Wildl. Manag..

[31] Godfrey SS, Moore JA, Nelson NJ, Bull CM (2010). Social network structure and parasite infection patterns in a territorial reptile, the tuatara (*Sphenodon punctatus*). Int. J. Parasitol..

[32] VanderWal E, Laforge MP, McLoughlin PD (2014). Density dependence in social behaviour: Home range overlap and density interacts to affect conspecific encounter rates in a gregarious ungulate. Behav. Ecol. Sociobiol..

[33] Lewis JS (2017). Contact networks reveal potential for interspecific interactions of sympatric wild felids driven by space use. Ecosphere.

[34] Brandell EE (2021). Group density, disease, and season shape territory size and overlap of social carnivores. J. Anim. Ecol..

[35] Krause J (2013). Reality mining of animal social systems. Trends Ecol. Evol..

[36] Fieberg J, Kochanny CO (2005). Quantifying home-range overlap: The importance of the utilization distribution. J. Wildl. Manag..

[37] Tilberg M, Dixon PM (2022). Statistical inference for the utilization distribution overlap index (UDOI). Methods Ecol. Evol..

[38] R Core Team. *R: A Language and Environment for Statistical Computing*. https://www.R-project.org/ (R Foundation for Statistical Computing, 2021).

[39] Calenge C (2006). The package adehabitat for the R software: Tool for the analysis of space and habitat use by animals. Ecol. Model..

[40] Fleming CH (2015). Rigorous home range estimation with movement data: A new autocorrelated kernel density estimator. Ecology.

[41] Silva I (2022). Autocorrelation-informed home range estimation: A review and practical guide. Methods Ecol. Evol..

[42] Farine DR, Whitehead H (2015). Constructing, conducting and interpreting animal social network analysis. J. Anim. Ecol..

[43] Sosa S, Sueur C, Puga-Gonzalez I (2021). Network measures in animal social network analysis: Their strengths, limits, interpretations and uses. Methods Ecol. Evol..

[44] White LA, Forester JD, Craft ME (2017). Using contact networks to explore mechanisms of parasite transmission in wildlife. Biol. Rev. Camb. Philos. Soc..

[45] Lloyd-Smith JO, Schreiber SJ, Kopp PE, Getz WM (2005). Superspreading and the effect of individual variation on disease emergence. Nature.

[46] Sah P, Leu ST, Cross PC, Hudson PJ, Bansal S (2017). Unraveling the disease consequences and mechanisms of modular structure in animal social networks. Proc. Natl. Acad. Sci. U.S.A..

[47] Croft DP, James R, Krause J (2008). Exploring Animal Social Networks.

[48] Csardi G, Nepusz T (2006). The igraph software package for complex network research. InterJournal.

[49] Sherman M, Cessie SL (1997). A comparison between bootstrap methods and generalized estimating equations for correlated outcomes in generalized linear models. Commun. Stat. Simul..

[50] National Oceanic and Atmospheric Administration. *Climate Data Online*. https://www.ncei.noaa.gov/cdo-web/.

[51] Albery GF, Kirkpatrick L, Firth JA, Bansal S (2021). Unifying spatial and social network analysis in disease ecology. J. Anim. Ecol..

[52] Lloyd-Smith JO (2009). Epidemic dynamics at the human–animal interface. Science.

[53] Sah P, Mann J, Bansal S (2018). Disease implications of animal social network structure: A synthesis across social systems. J. Anim. Ecol..

[54] Hamede R, Bashford J, Jones M, McCallum H (2012). Simulating devil facial tumour disease outbreaks across empirically derived contact networks. J. Appl. Ecol..

[55] Marino S, Hogue IB, Ray CJ, Kirschner DE (2008). A methodology for performing global uncertainty and sensitivity analysis in systems biology. J. Theor. Biol..

[56] Delignette-Muller ML, Dutang C (2015). fitdistrplus: An R package for fitting distributions. J. Stat. Softw..

[57] Hunter DR, Handcock MS, Butts CT, Goodreau SM, Morris M (2008). ergm: A package to fit, simulate and diagnose exponential-family models for networks. J. Stat. Softw..

[58] Davis GH, Crofoot MC, Farine DR (2018). Estimating the robustness and uncertainty of animal social networks using different observational methods. Anim. Behav..

[59] Silk MJ, Jackson AL, Croft DP, Colhoun K, Bearhop S (2015). The consequences of unidentifiable individuals for the analysis of an animal social network. Anim. Behav..

[60] Gilbertson MLJ, White LA, Craft ME (2020). Trade-offs with telemetry-derived contact networks for infectious disease studies in wildlife. Methods Ecol. Evol..

[61] Liaw A, Wiener M (2002). Classification and regression by randomForest. R News..

[62] White LA, VandeWoude S, Craft ME (2020). A mechanistic, stigmergy model of territory formation in solitary animals: Territorial behavior can dampen disease prevalence but increase persistence. PLoS Comput. Biol..

[63] Bolker BM (2008). Ecological Models and Data in R.

[64] Hostetler JA (2012). Does genetic introgression improve female reproductive performance? A test on the endangered Florida panther. Oecologia.

[65] Cunningham MW (2021). Pseudorabies (Aujeszky’s disease) is an underdiagnosed cause of death in the Florida panther (*Puma concolor coryi*). J. Wildl. Dis..

[66] Zeller KA, Vickers TW, Ernest HB, Boyce WM (2017). Multi-level, multi-scale resource selection functions and resistance surfaces for conservation planning: Pumas as a case study. PLoS ONE.

[67] Hopcraft JGC, Sinclair ARE, Packer C (2005). Planning for success: Serengeti lions seek prey accessibility rather than abundance. J. Anim. Ecol..

[68] Wilber MQ (2022). A model for leveraging animal movement to understand spatio-temporal disease dynamics. Ecol. Lett..

[69] Manlove K (2022). Defining an epidemiological landscape that connects movement ecology to pathogen transmission and pace-of-life. Ecol. Lett..

[70] Allen ML (2015). The role of scent marking in mate selection by female pumas (*Puma concolor*). PLoS ONE.

[71] Elbroch LM, Levy M, Lubell M, Quigley H, Caragiulo A (2017). Adaptive social strategies in a solitary carnivore. Sci. Adv..

[72] Obeysekera J, Barnes J, Nungesser M (2015). Climate sensitivity runs and regional hydrologic modeling for predicting the response of the greater Florida Everglades ecosystem to climate change. Environ. Manag..

[73] Miller ER, Fowler ME (2014). Fowler’s Zoo and Wild Animal Medicine.

[74] Gremillion-Smith C, Woolf A (1988). Epizootiology of skunk rabies in North America. J. Wildl. Dis..

[75] Altizer S, Hochachka WM, Dhondt AA (2004). Seasonal dynamics of mycoplasmal conjunctivitis in eastern North American house finches. J. Anim. Ecol..

[76] Dhondt AA, States SL, Dhondt KV, Schat KA (2012). Understanding the origin of seasonal epidemics of mycoplasmal conjunctivitis. J. Anim. Ecol..

[77] Swinton J (1998). Persistence thresholds for phocine distemper virus infection in harbour seal *Phoca vitulina* metapopulations. J. Anim. Ecol..

[78] Duignan PJ (2014). Phocine distemper virus: Current knowledge and future directions. Viruses.

[79] Hamede RK, McCallum H, Jones M (2013). Biting injuries and transmission of Tasmanian devil facial tumour disease. J. Anim. Ecol..

[80] Hamede RK, Bashford J, McCallum H, Jones M (2009). Contact networks in a wild Tasmanian devil (*Sarcophilus harrisii*) population: Using social network analysis to reveal seasonal variability in social behaviour and its implications for transmission of devil facial tumour disease. Ecol. Lett..

[81] Hamede RK, Mccallum H, Jones M (2008). Seasonal, demographic and density-related patterns of contact between Tasmanian devils (*Sarcophilus harrisii*): Implications for transmission of devil facial tumour disease. Austral Ecol..

[82] McCallum H (2009). Transmission dynamics of Tasmanian devil facial tumor disease may lead to disease-induced extinction. Ecology.

[83] Gustafson KD, Vickers TW, Boyce WM, Ernest HB (2017). A single migrant enhances the genetic diversity of an inbred puma population. R. Soc. Open Sci..

[84] Carlson CJ (2022). Climate change increases cross-species viral transmission risk. Nature.

[85] Hostetler JA, Onorato DP, Jansen D, Oli MK (2013). A cat’s tale: The impact of genetic restoration on Florida panther population dynamics and persistence. J. Anim. Ecol..

[86] Martin LB (2019). Extreme competence: Keystone hosts of infections. Trends Ecol. Evol..

[87] Guerra MA (2003). Skunk and raccoon rabies in the eastern United States: Temporal and spatial analysis. Emerg. Infect. Dis..

