# Supplementary material for: Seasonal changes in network connectivity and consequences for pathogen transmission in a solitary carnivore
Source: Sci Rep. 2023 Oct 18;13:17802. doi: 10.1038/s41598-023-44815-y (PMC10584909; doi:10.1038/s41598-023-44815-y)
Supplement: Supplementary file 1 — Supplementary Information. [file 41598_2023_44815_MOESM1_ESM.docx]

**Supplementary material for: Seasonal changes in network connectivity and consequences for pathogen transmission in a solitary carnivore**

Marie L.J. Gilbertson^1†^*, S. Niamh Hart^1^, Kimberly VanderWaal^1^, Dave Onorato^2^, Mark Cunningham^3^, Sue VandeWoude^4^, & Meggan E. Craft^1,5^

*Corresponding author: mgilbertson5@wisc.edu

^1^Department of Veterinary Population Medicine, University of Minnesota, St Paul, MN 55108.

^2^Fish and Wildlife Research Institute, Florida Fish and Wildlife Conservation Commission, Naples, FL 34114.

^3^Fish and Wildlife Research Institute, Florida Fish and Wildlife Conservation Commission, Gainesville, FL 32601.

^4^Department of Microbiology, Immunology, and Pathology, Colorado State University, Fort Collins, CO 80523

^5^Department of Ecology, Evolution and Behavior, University of Minnesota, St Paul, MN 55108.

^†^Current affiliation: Wisconsin Cooperative Wildlife Research Unit, Department of Forest and Wildlife Ecology, University of Wisconsin–Madison, Madison, WI, 53706

**
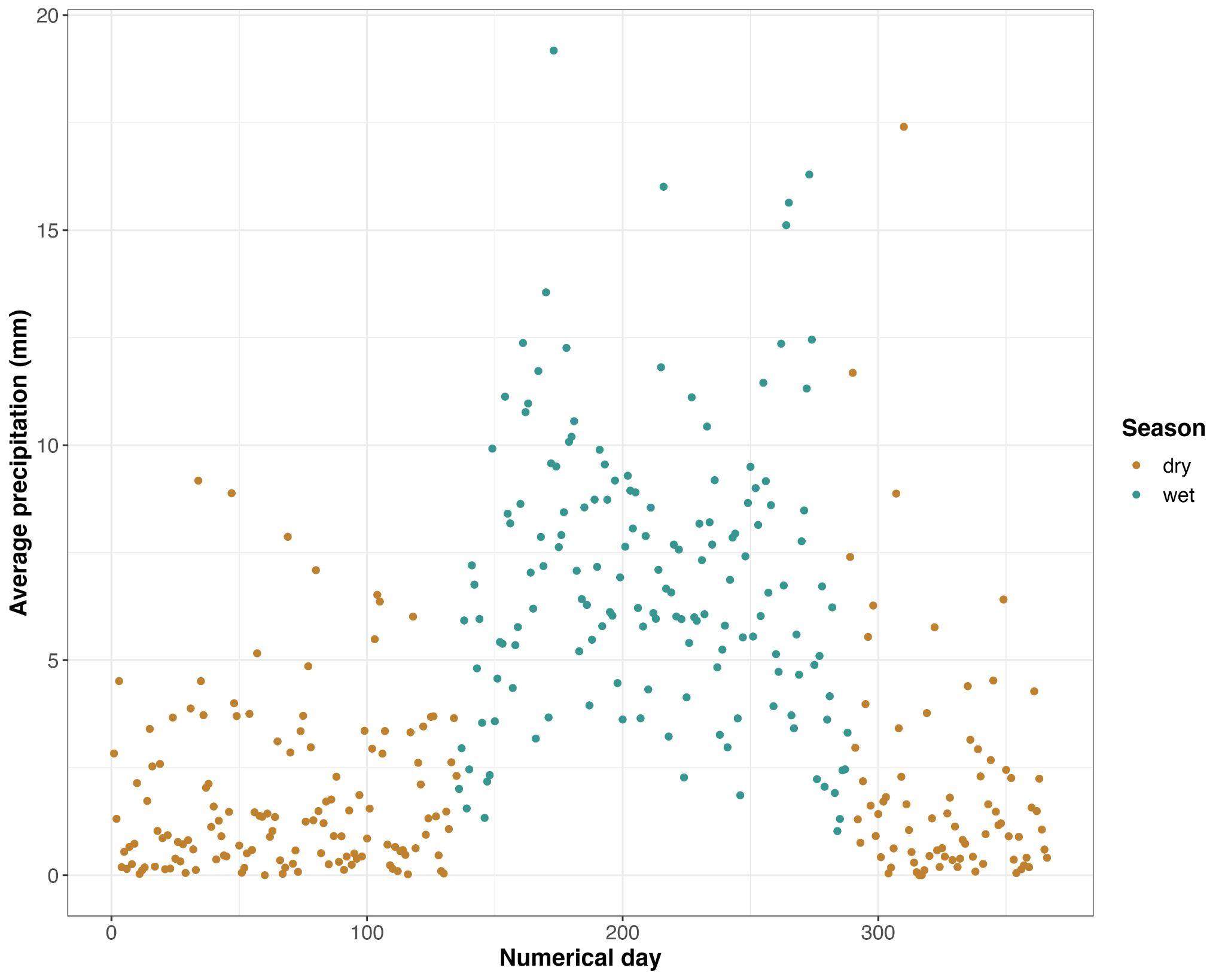
**

**Figure S1:** Annual average daily precipitation (in mm) in Collier county, Florida. The x-axis shows numerical or Julian day (e.g., day 15 = January 15). Points are colored by season as defined in our analyses (wet season = 15 May through 14 October; dry season = 15 October through 14 May). To calculate daily precipitation averages for this visualization of south Florida seasonality, we averaged precipitation amounts across Collier county weather stations for each day in our study period (1996-2007), then took the average rainfall across years for each numerical day (e.g., averaged the average rainfall amounts for January 15 from 1996-2007).

**
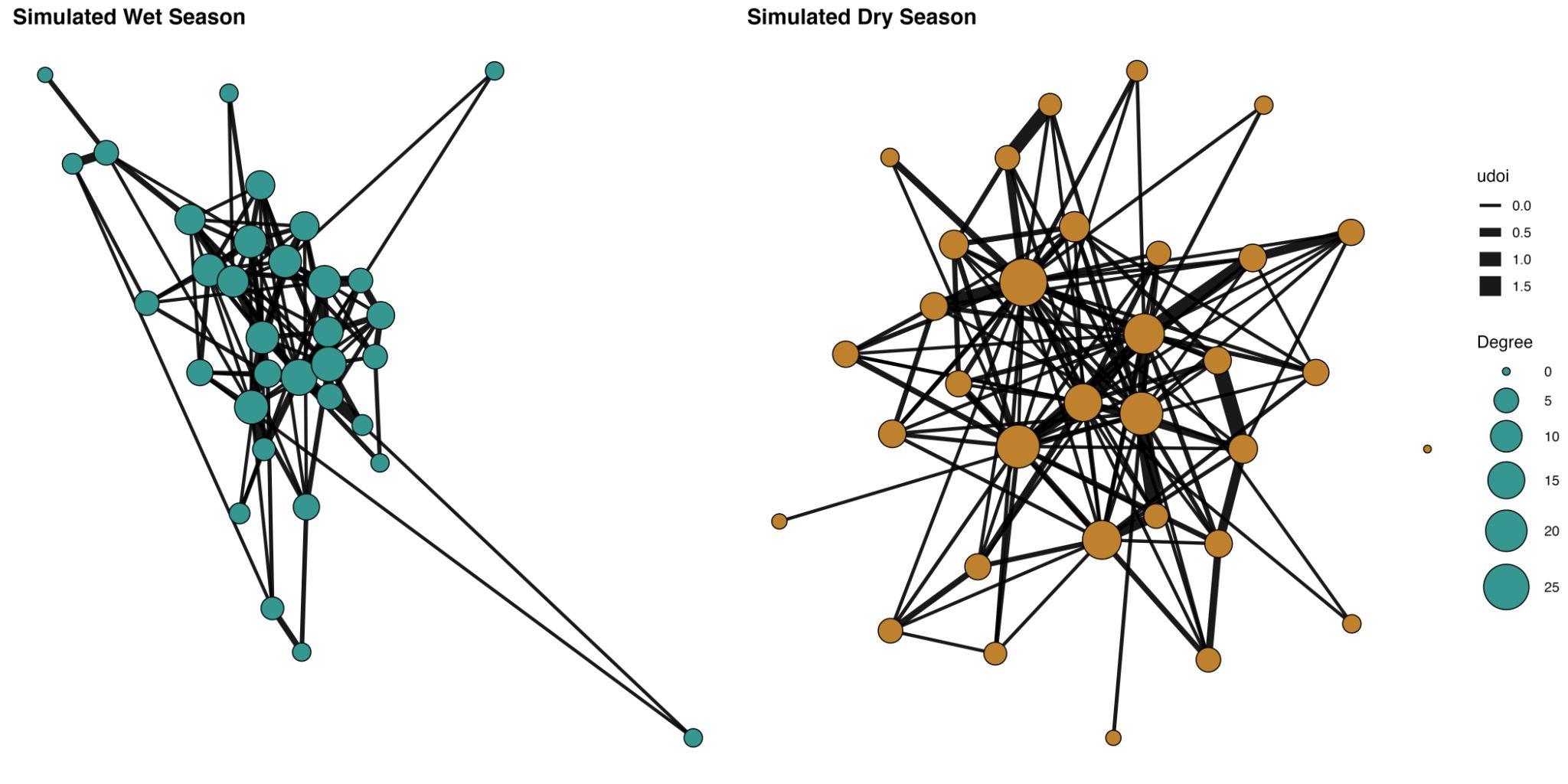
**

**Figure S2:** Representative examples of simulated wet (left) and dry (right) season networks. Node sizes are relative to degree (a higher number of connections results in a higher node size) and edge thickness corresponds to utilization distribution overlap index (UDOI)

**Seasonal panther networks**

Panther home range overlap networks are given in figures S1-S11. Each figure gives the wet and dry season networks for a given year. Network nodes have a Fruchterman Reingold layout for visibility (i.e., node position does not correspond to geographic location) and are colored by season (wet season = aqua blue, dry season = dark tan). Node sizes are relative to degree (a higher number of connections results in a higher node size) and edge thickness corresponds to utilization distribution overlap index (UDOI), with higher overlap corresponding to thicker edge weights.

**
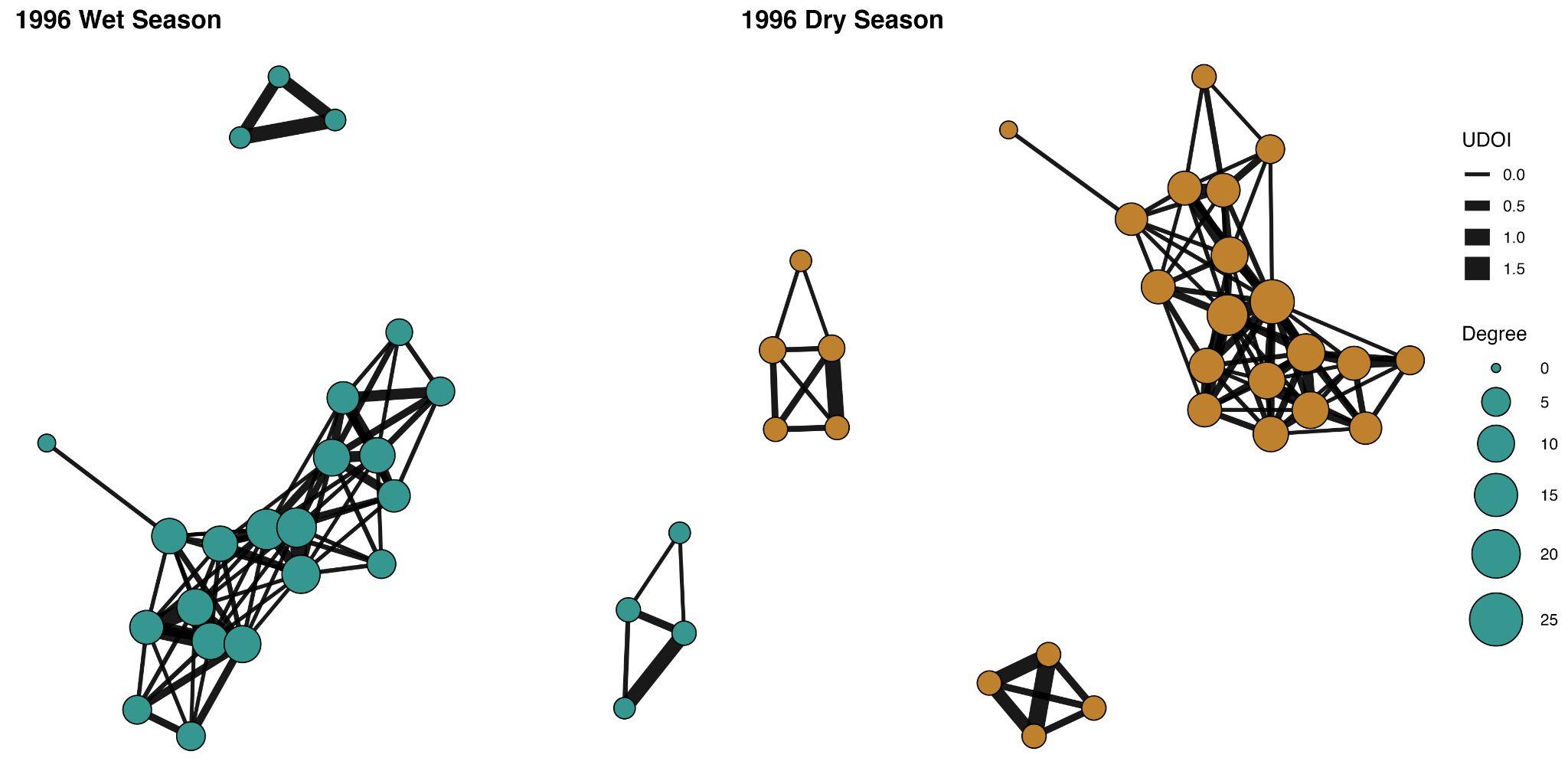
**

**Figure S3:** 1996 Florida panther overlap networks

**
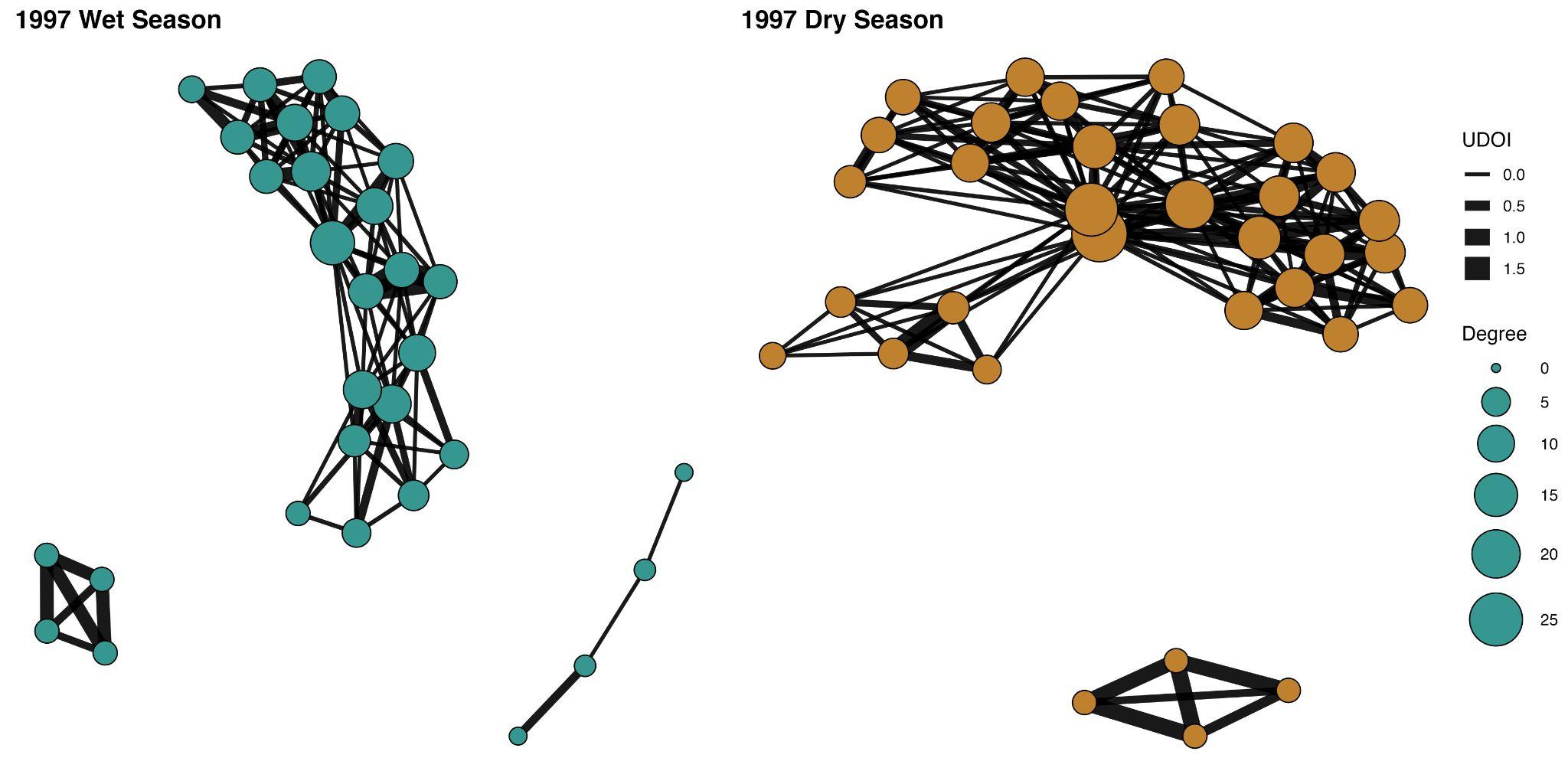
**

**Figure S4:** 1997 Florida panther overlap networks

**
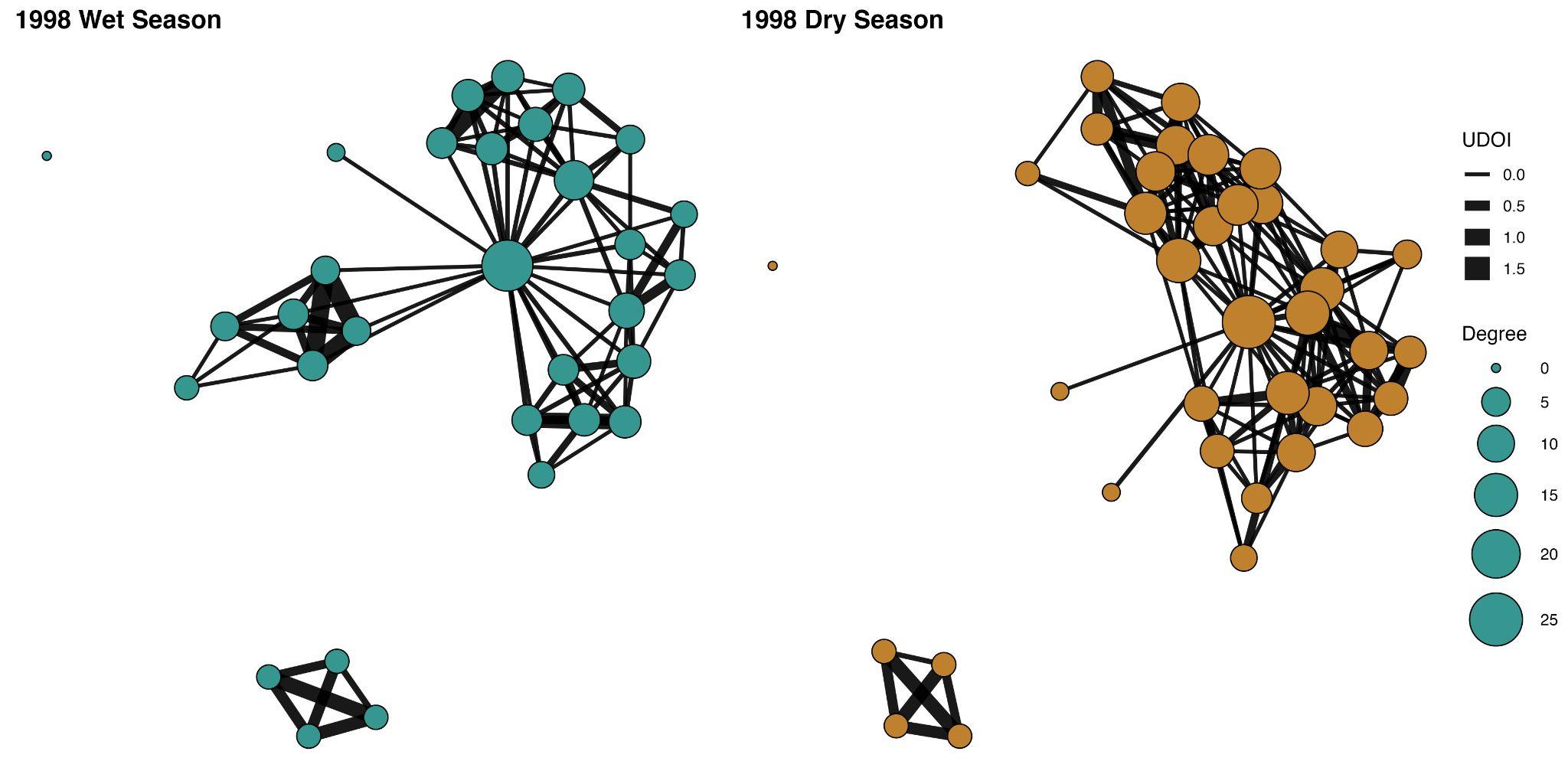
**

**Figure S5:** 1998 Florida panther overlap networks

**
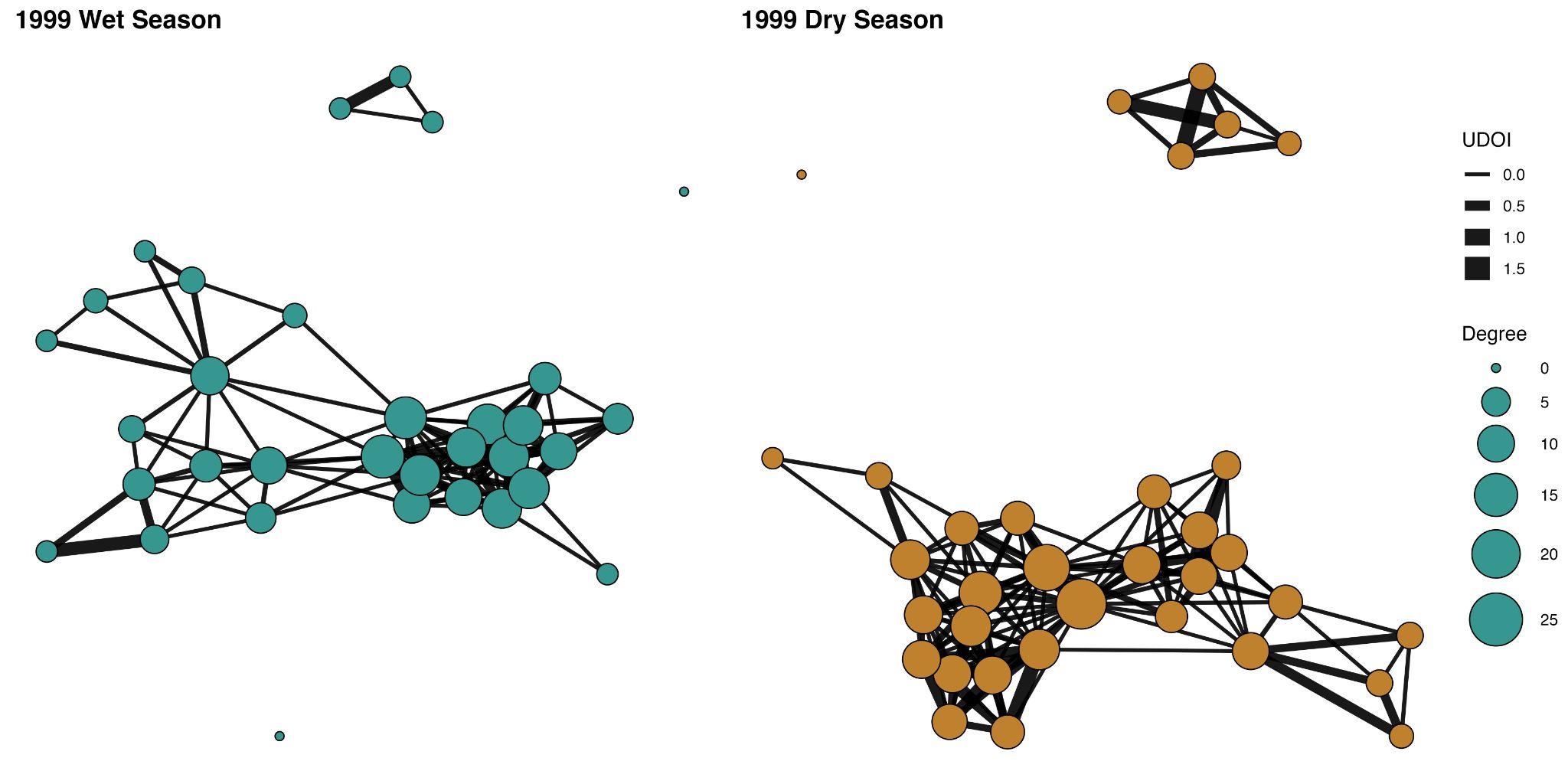
**

**Figure S6:** 1999 Florida panther overlap networks

**
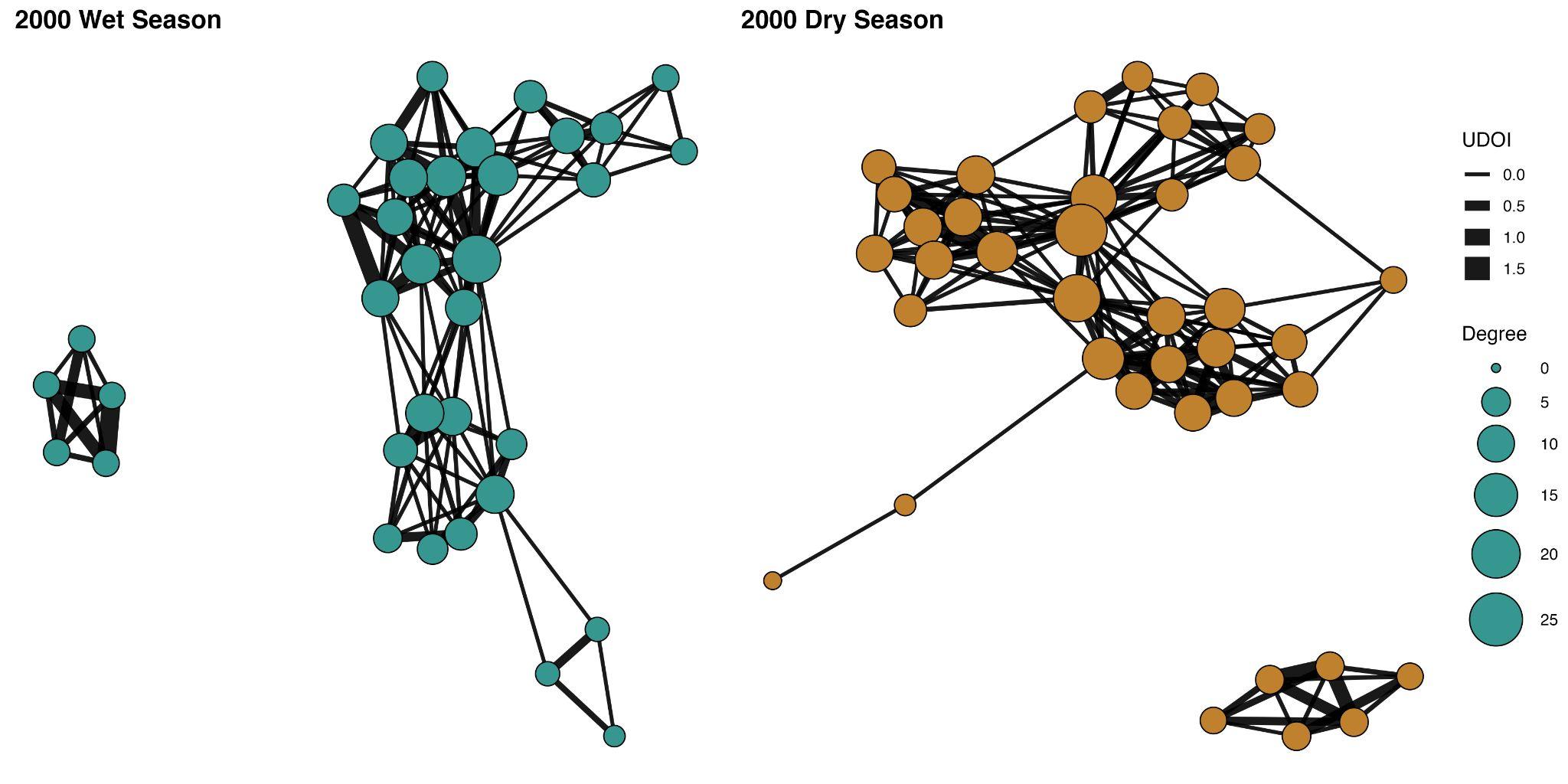
**

**Figure S7:** 2000 Florida panther overlap networks

**
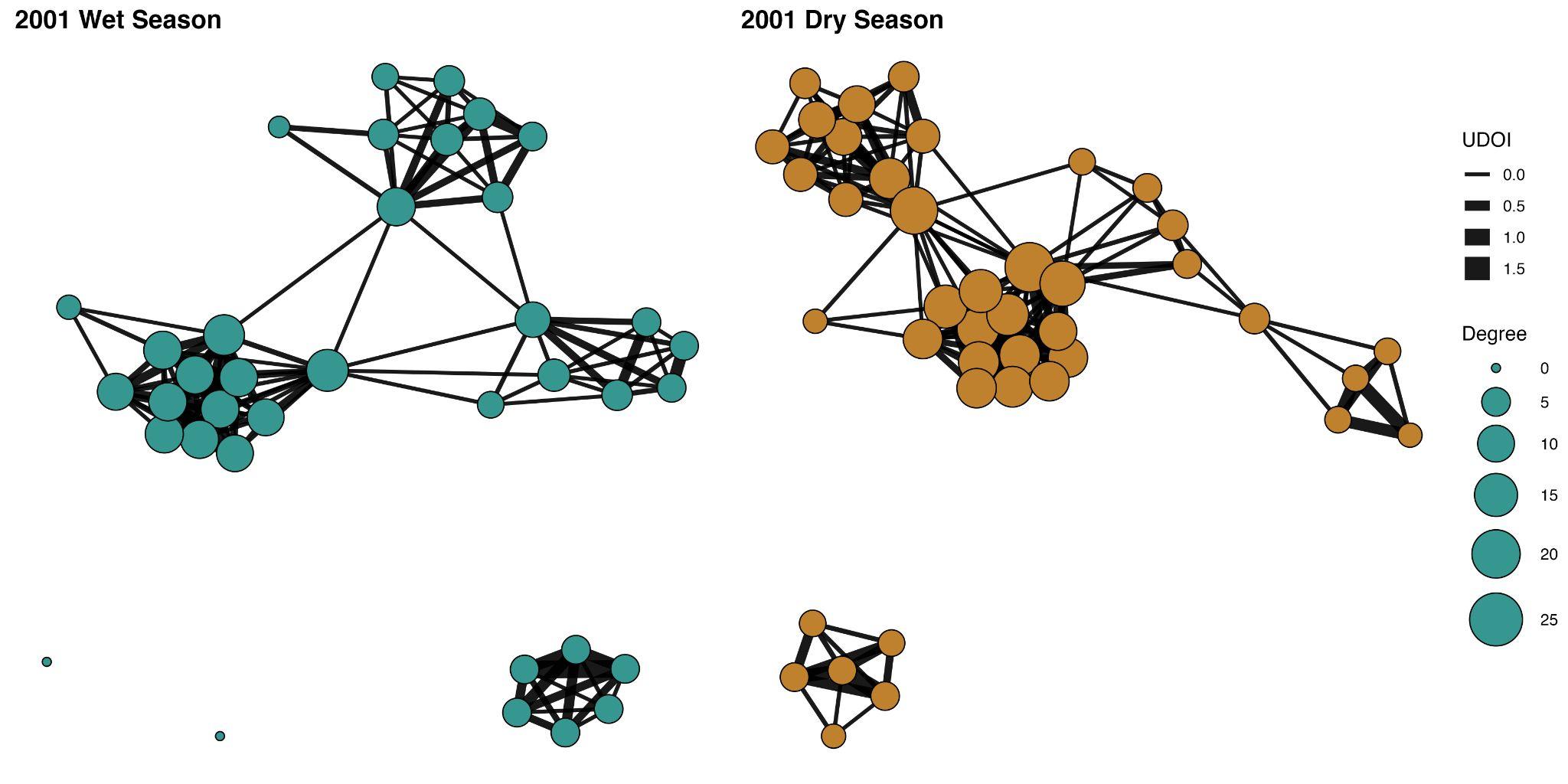
**

**Figure S8:** 2001 Florida panther overlap networks

**
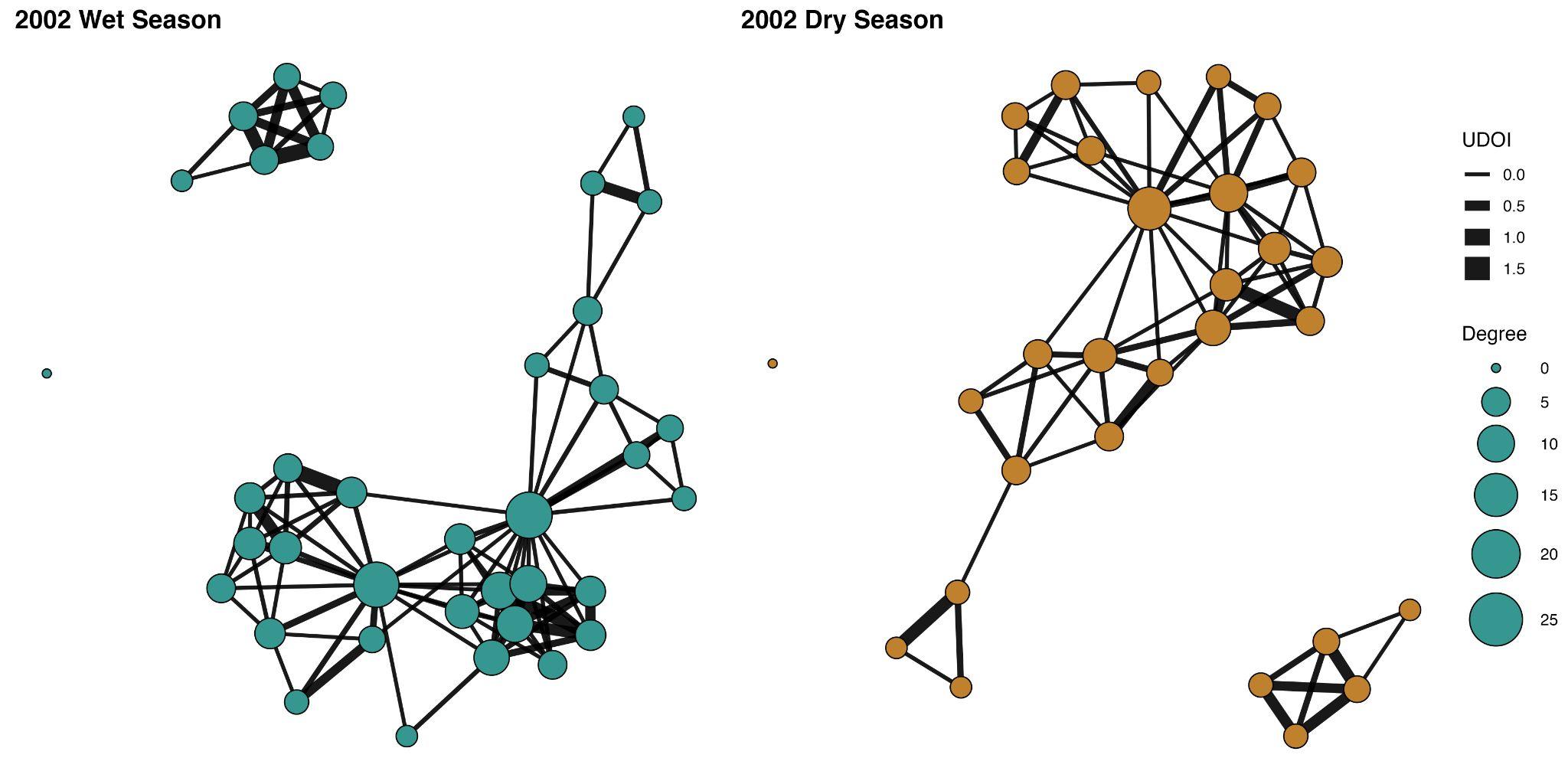
**

**Figure S9:** 2002 Florida panther overlap networks

**
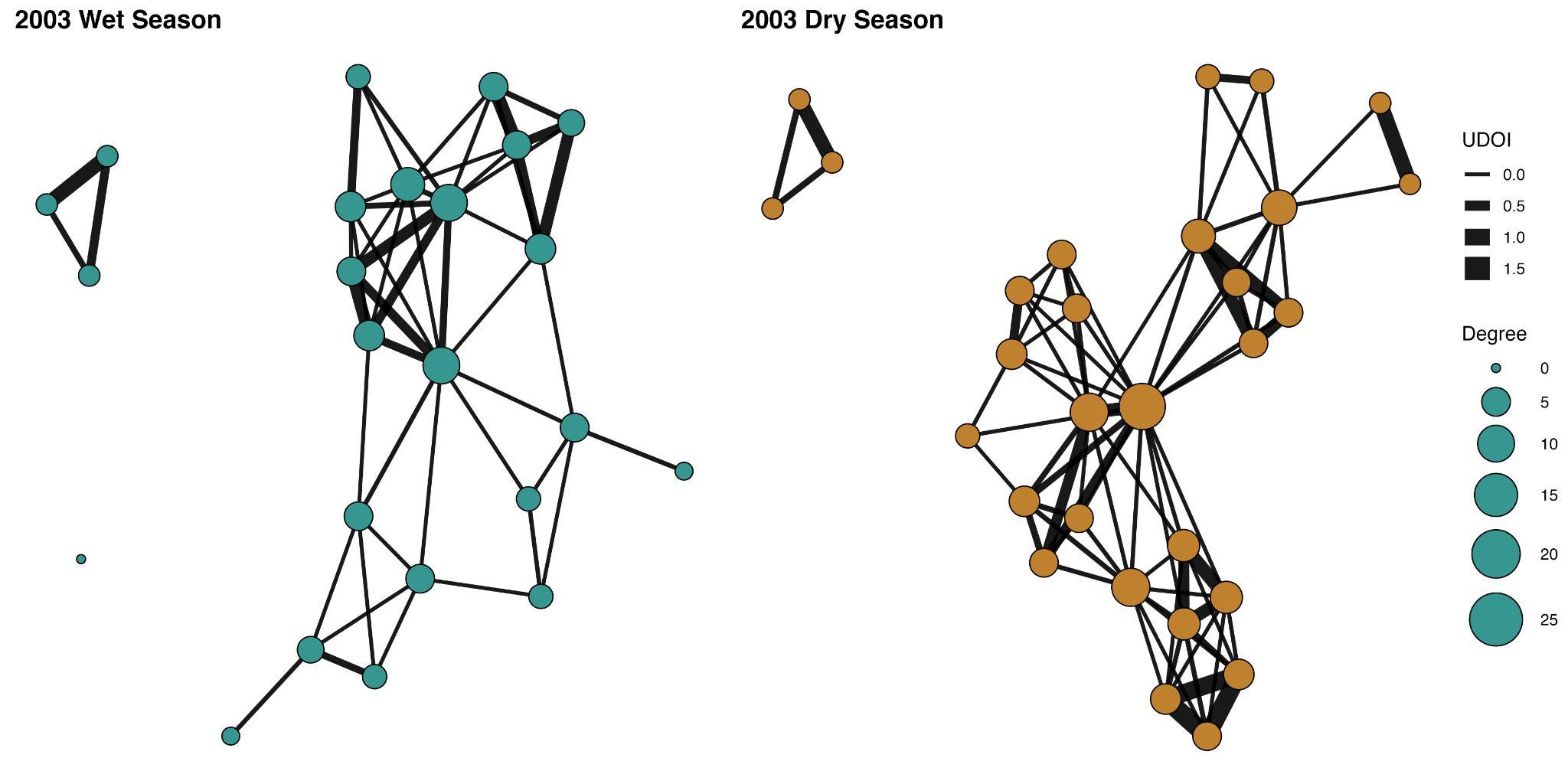
**

**Figure S10:** 2003 Florida panther overlap networks

**
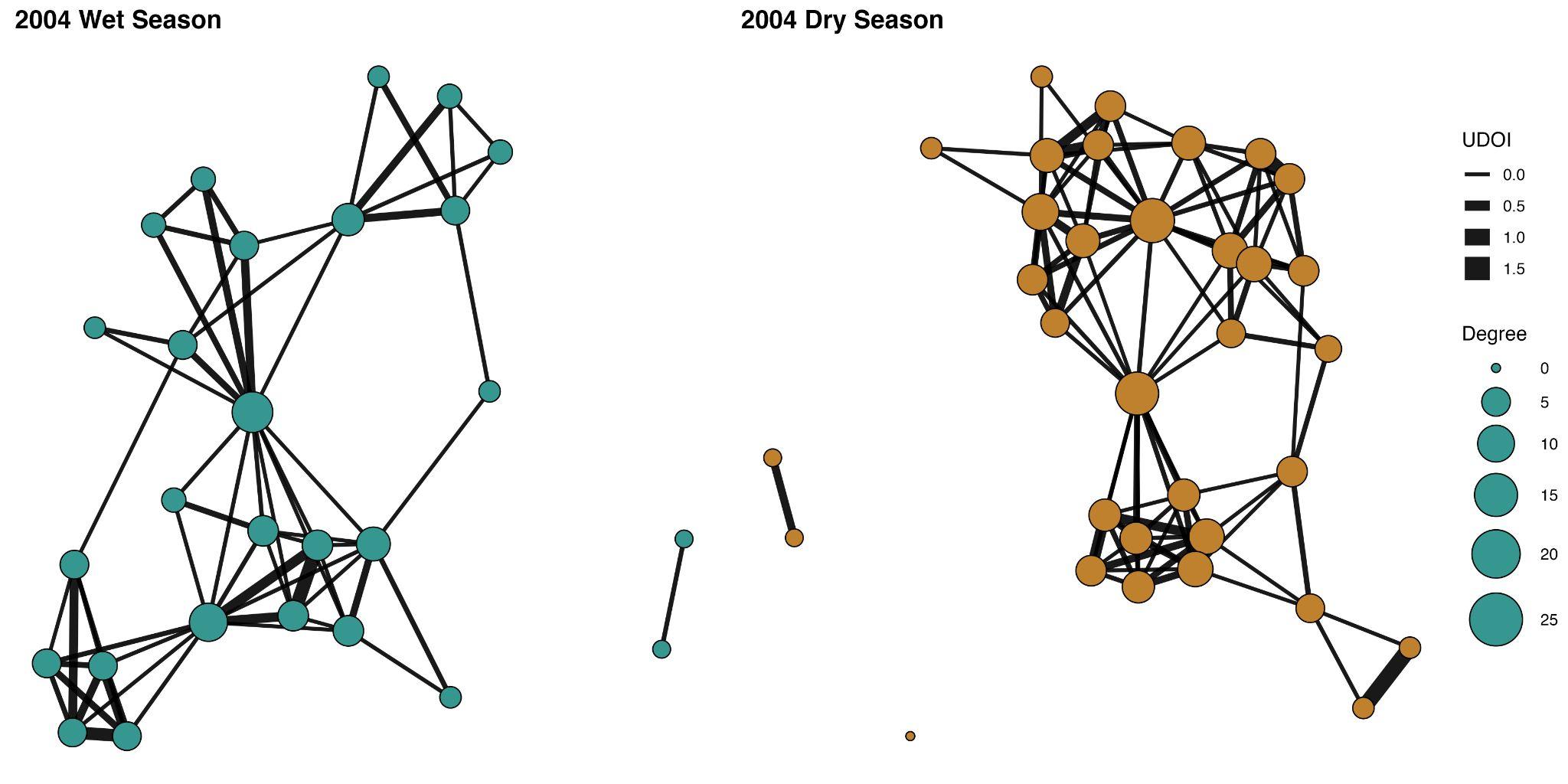
**

**Figure S11:** 2004 Florida panther overlap networks

**
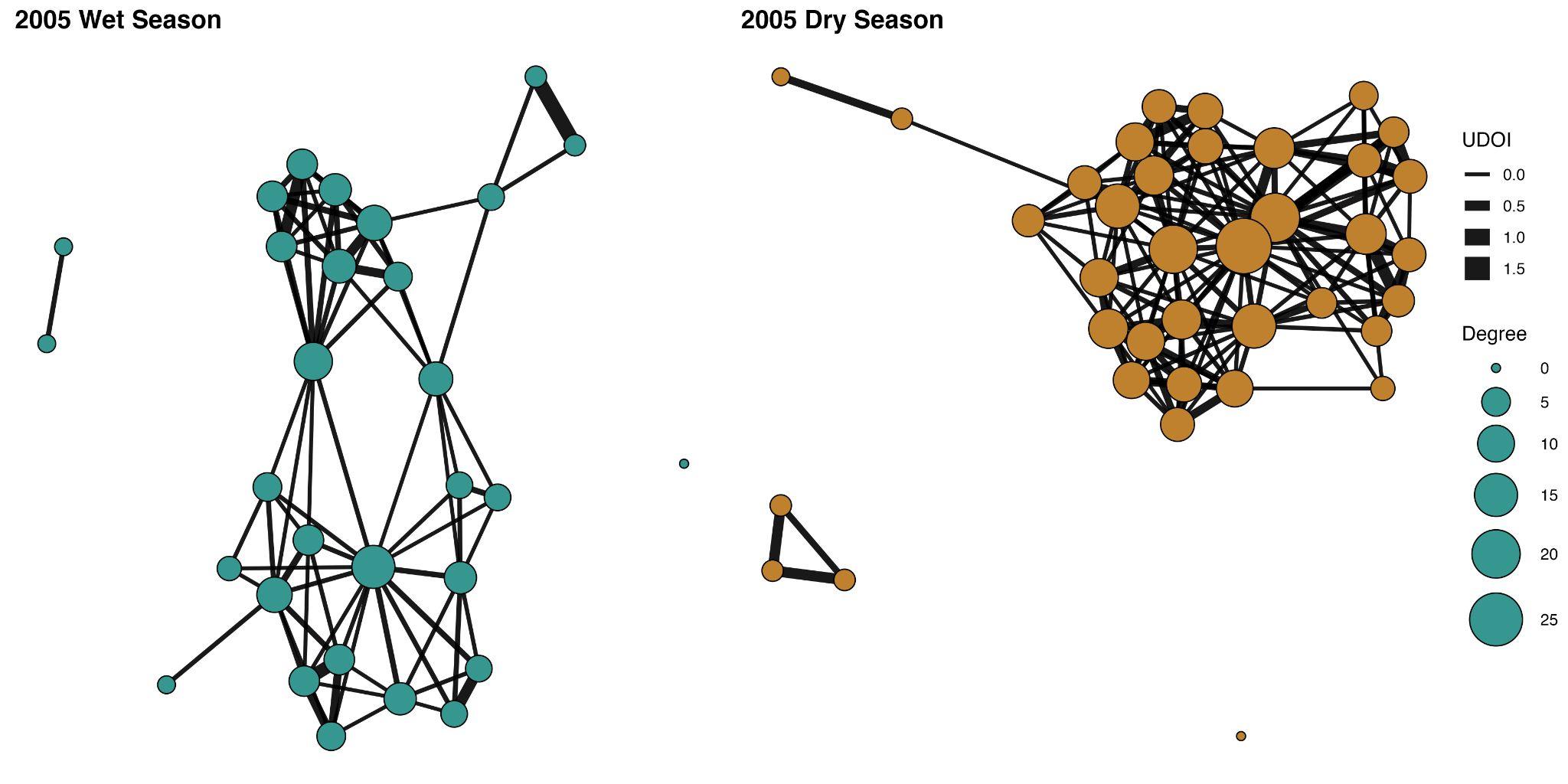
**

**Figure S12:** 2005 Florida panther overlap networks

**
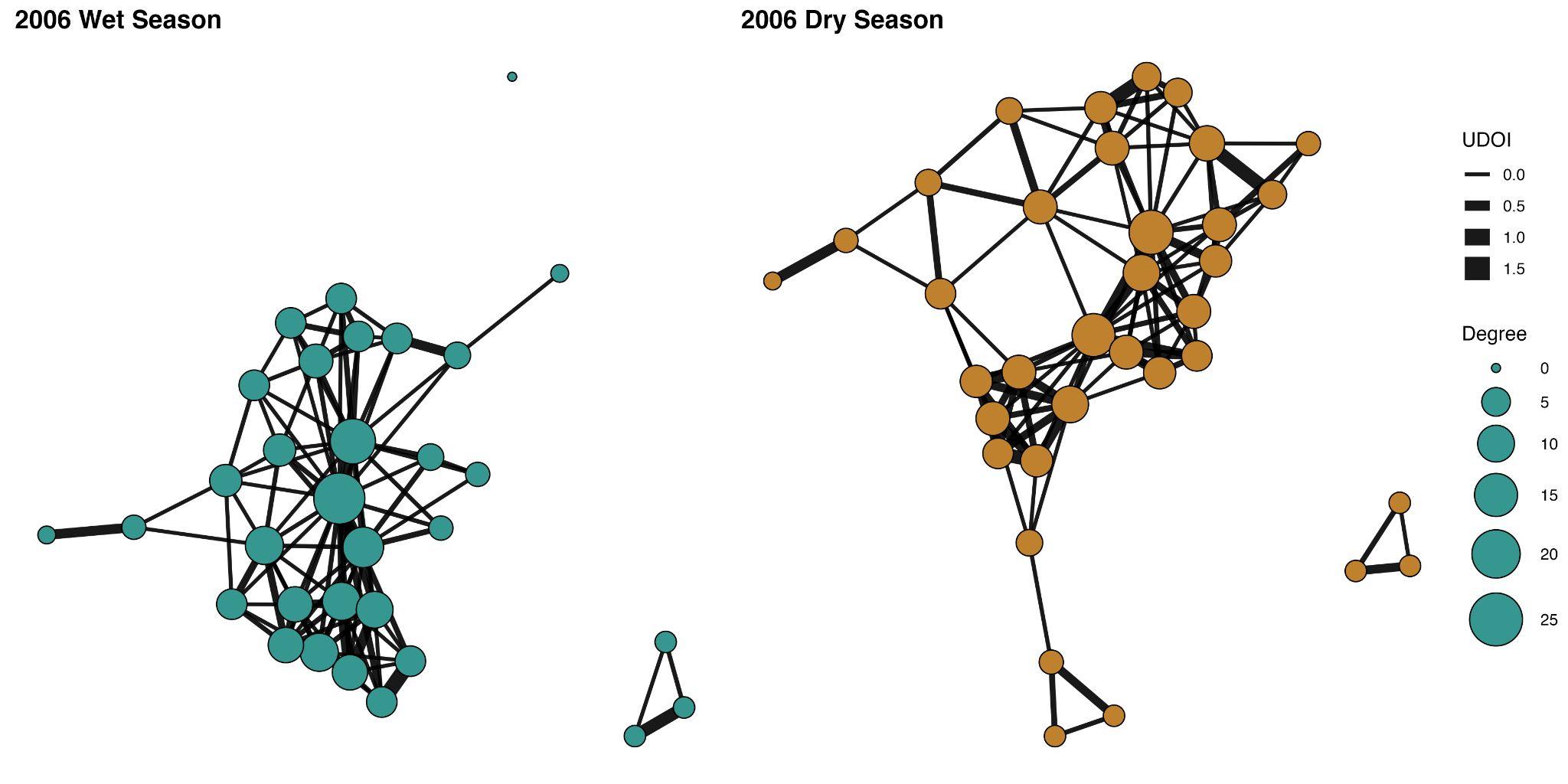
**

**Figure S13:** 2006 Florida panther overlap networks

**Table S1: Panther sample size and median home range overlap across years and seasons**

|  | **Panthers (n = )** | | **Median UDOI (all)** | | **Median UDOI (>0.1)** | |
| --- | --- | --- | --- | --- | --- | --- |
| **Year** | **Wet** | **Dry** | **Wet** | **Dry** | **Wet** | **Dry** |
| 1996 | 26 | 32 | 0.06 | 0.13 | 0.41 | 0.25 |
| 1997 | 30 | 36 | 0.05 | 0.05 | 0.29 | 0.26 |
| 1998 | 33 | 37 | 0.07 | 0.03 | 0.30 | 0.24 |
| 1999 | 33 | 47 | 0.01 | 0.04 | 0.25 | 0.27 |
| 2000 | 37 | 45 | 0.02 | 0.02 | 0.27 | 0.24 |
| 2001 | 37 | 43 | 0.10 | 0.04 | 0.30 | 0.26 |
| 2002 | 39 | 40 | 0.03 | 0.03 | 0.28 | 0.22 |
| 2003 | 31 | 38 | 0.02 | 0.03 | 0.40 | 0.54 |
| 2004 | 31 | 36 | 0.03 | 0.06 | 0.27 | 0.19 |
| 2005 | 32 | 39 | 0.04 | 0.03 | 0.17 | 0.25 |
| 2006 | 34 | 38 | 0.02 | 0.08 | 0.20 | 0.26 |

*Note: The “Panthers (n = )” columns give sample sizes of panthers for each year and season. UDOI = utilization distribution overlap index, a quantification of home range overlap. The UDOI (all) columns give median UDOI values across all pairs of overlapping individuals. The UDOI (>0.1) columns give median UDOI values for the subset of pairs with UDOI values greater than 0.1.*

**
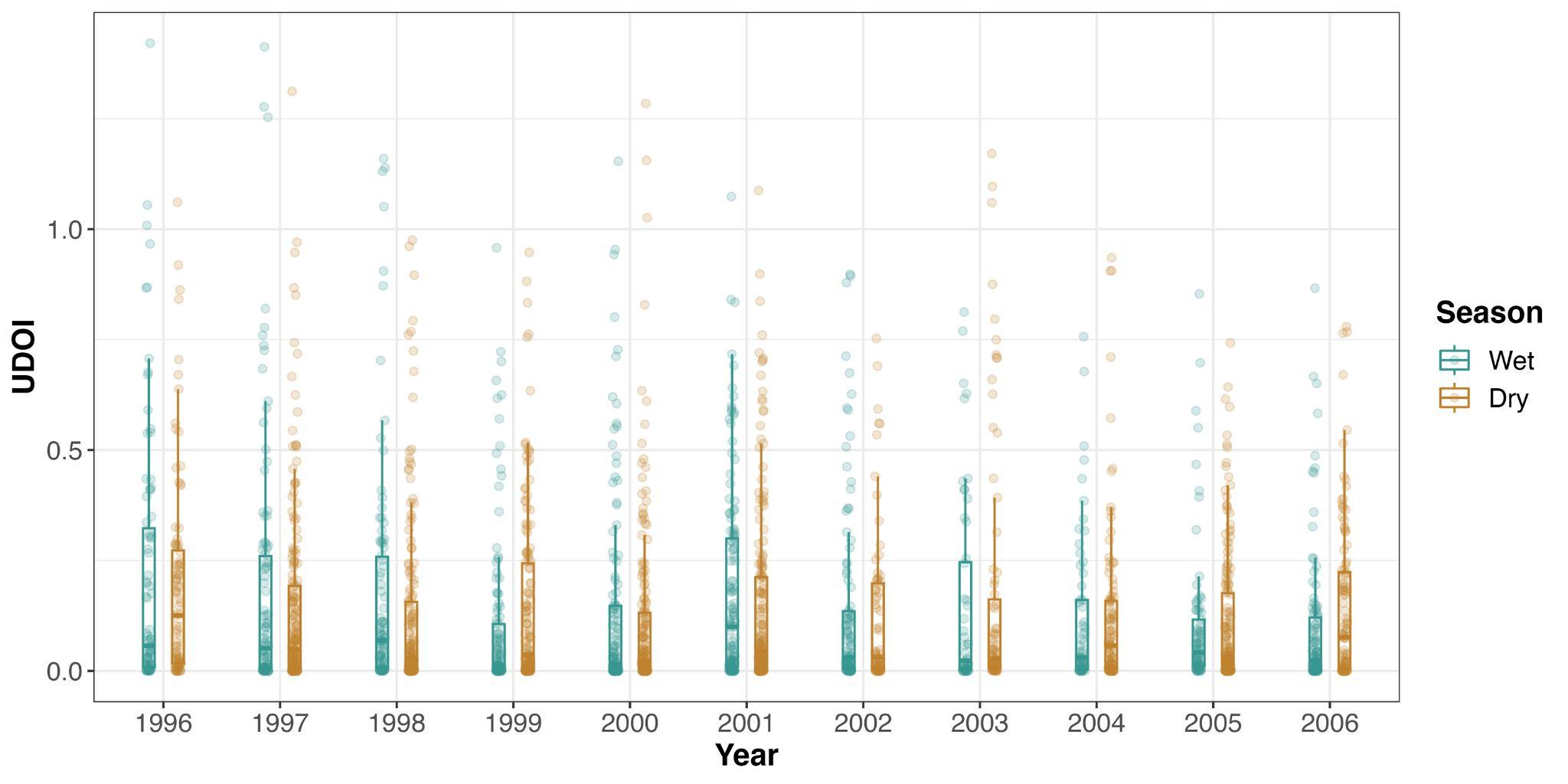
**

**Figure S14:** Utilization distribution overlap index (UDOI) values for all pairs of overlapping panthers by year and season. Boxplots highlight the right-skewed distributions of UDOI values.


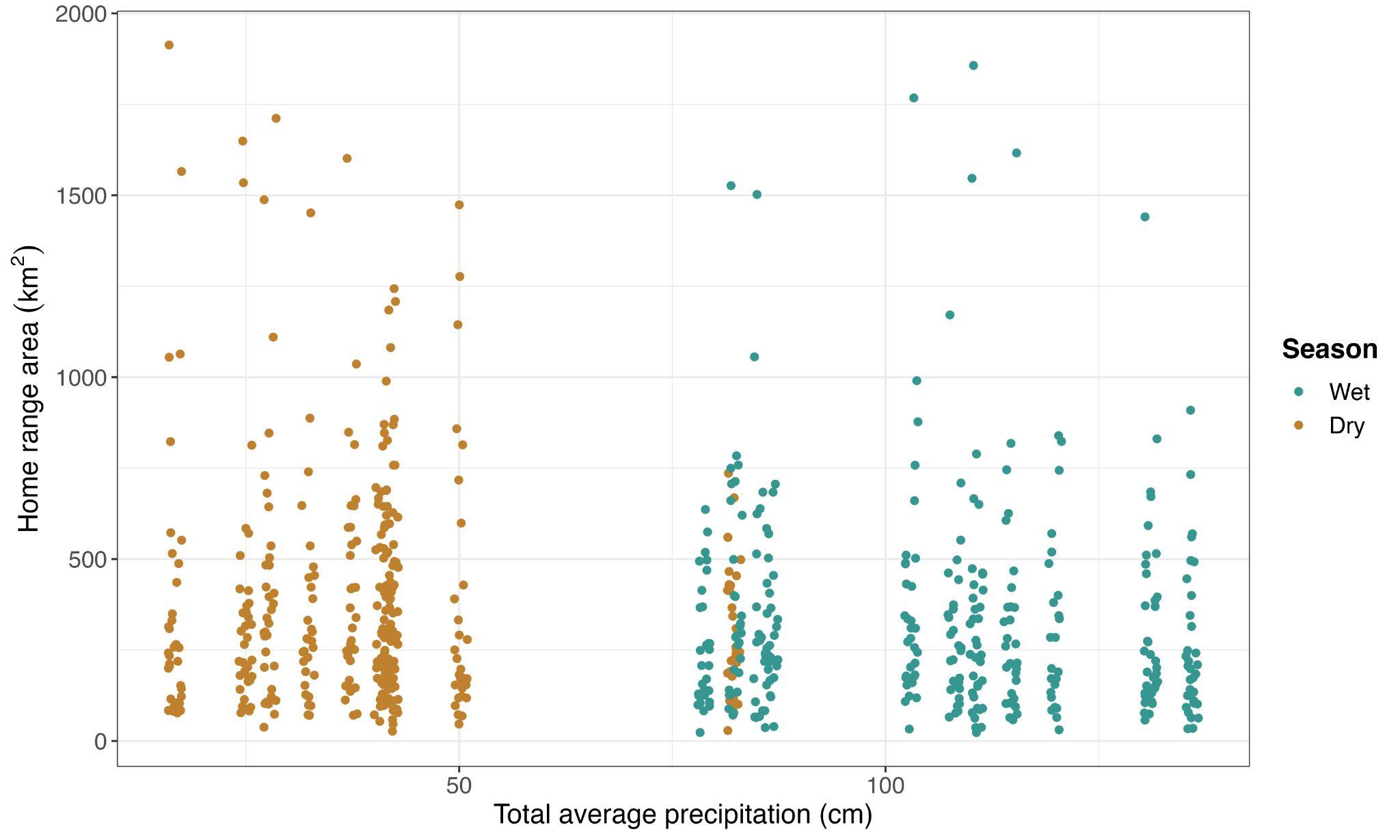


**Figure S15:** Panther home range area (in km^2^) compared to total average seasonal precipitation (in cm). Points represent the home range size of a single panther in a given season (aqua blue = wet season, dark tan = dry season). Note that outlier home ranges were excluded for this visualization (home ranges that were larger than two standard deviations greater than the mean home range size).


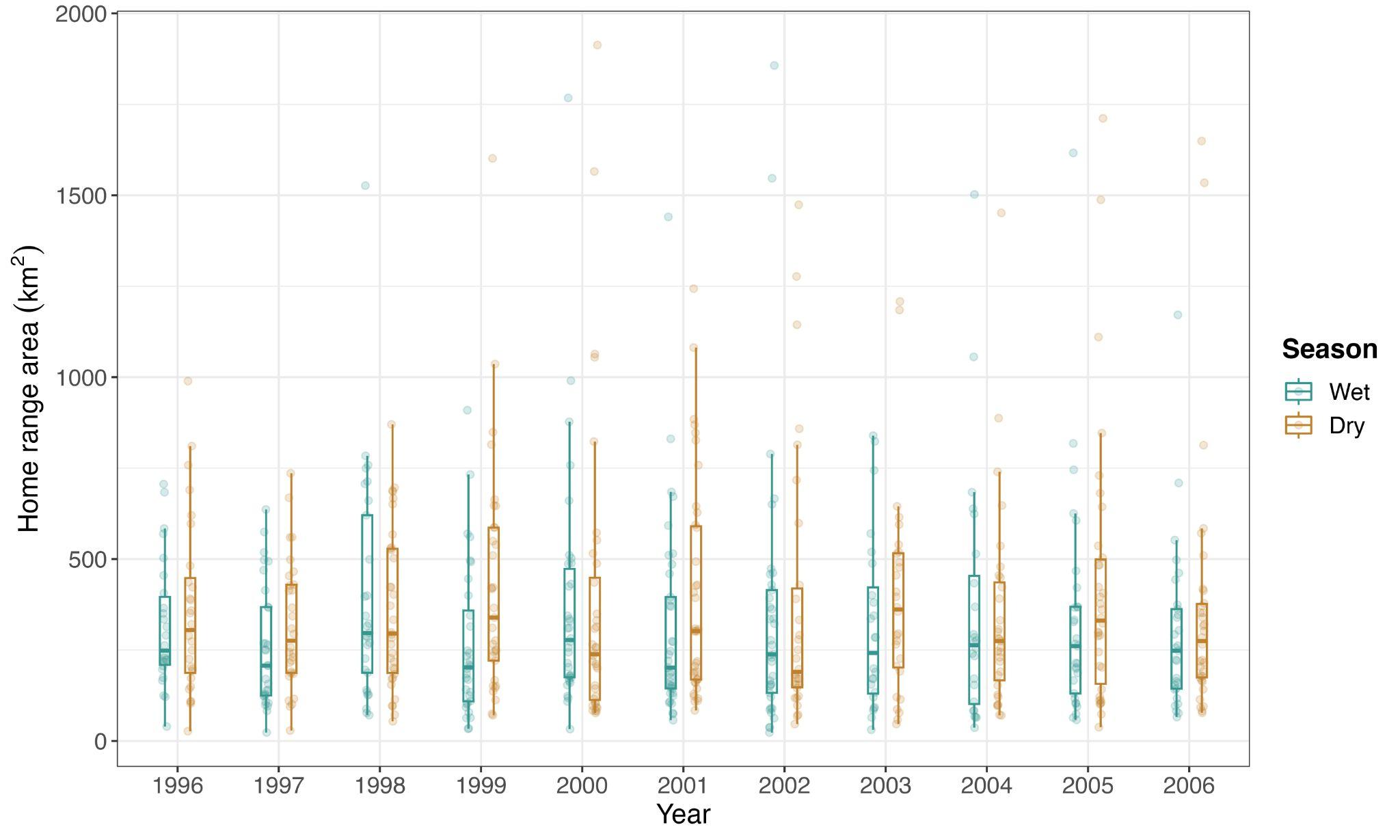


**Figure S16:** Panther home range area (in km^2^) by year and season (aqua blue = wet season, dark tan = dry season). Points represent the home range size of a single panther in a given season. Note that outlier home ranges were excluded for this visualization (home ranges that were larger than two standard deviations greater than the mean home range size).

**Table S2: Cluster level bootstrap results across network edge filtering levels**

| **UDOI filter** | **Node Metric** | **Variable** | **Estimate** | **Coefficient 95% CI** |
| --- | --- | --- | --- | --- |
| 0 | Normalized degree | Intercept | -0.1 | (-0.2, 0.007) |
|  |  | **Log(HR area)** | **0.06** | **(0.04, 0.08)*** |
|  |  | **Season (Wet)** | **-0.02** | **(-0.03, -0.006)*** |
|  | Strength | Intercept | 0.04 | (-0.4, 0.5) |
|  |  | **Log(HR area)** | **0.2** | **(0.09, 0.3)*** |
|  |  | **Season (Wet)** | **-0.1** | **(-0.2, -0.08)*** |
| 0.01 | Normalized degree | Intercept | -0.06 | (-0.1, 0.01) |
|  |  | **Log(HR area)** | **0.04** | **(0.03, 0.05)*** |
|  |  | **Season (Wet)** | **-0.02** | **(-0.03, -0.01)*** |
|  | Strength | Intercept | 0.04 | (-0.4, 0.5) |
|  |  | **Log(HR area)** | **0.2** | **(0.1, 0.3)*** |
|  |  | **Season (Wet)** | **-0.1** | **(-0.2, -0.08)*** |
| 0.1 | Normalized degree | Intercept | -0.02 | (-0.06, 0.03) |
|  |  | **Log(HR area)** | **0.02** | **(0.01, 0.02)*** |
|  |  | **Season (Wet)** | **-0.01** | **(-0.01, -0.006)*** |
|  | Strength | Intercept | 0.1 | (-0.4, 0.5) |
|  |  | **Log(HR area)** | **0.1** | **(0.07, 0.2)*** |
|  |  | **Season (Wet)** | **-0.1** | **(-0.2, -0.06)*** |

*Note: Linear model coefficient estimates with 95% confidence intervals (CI) from cluster level bootstrap analysis across UDOI filtering levels. UDOI filter indicates level of home range overlap between individuals to count as an edge (e.g., UDOI > 0). Home range area was modeled as the log of the home range area in square km; dry season was the reference level for season, so coefficient estimates represent the effect of wet season on the respective node level metric. CIs that do not cross zero are considered statistically significant (marked by * and bold text).*

**Table S3: Spearman correlation for median node-level metrics and precipitation**

| **Metric** | **Season** | **Spearman’s rho** | ***p*-value** |
| --- | --- | --- | --- |
| Normalized degree | Dry | 0.09 | 0.80 |
|  | Wet | -0.41 | 0.21 |
| Strength | Dry | 0.36 | 0.27 |
|  | Wet | -0.35 | 0.30 |

*Note: Correlations were between median metric per season/year and total precipitation values per season/year (see main text).*

**Table S4: Kruskal-Wallis rank sum test results across network edge filtering levels**

| **UDOI filter** | **Network metric** | **Rank Sum** | ***p*-value** |
| --- | --- | --- | --- |
| 0 | Density | 1.99 | 0.16 |
|  | Modularity (4 steps) | 3.75 | 0.053 |
|  | Modularity (7 steps) | 3.75 | 0.053 |
| 0.01 | Density | 3.26 | 0.071 |
|  | Modularity (4 steps) | 0.001 | 0.97 |
|  | Modularity (7 steps) | 0.001 | 0.97 |
| 0.1 | Density | 2.18 | 0.14 |
|  | Modularity (4 steps) | 0.01 | 0.92 |
|  | Modularity (7 steps) | 0.001 | 0.97 |

*Note: Kruskal-Wallis rank sum test for differences in network metrics between wet and dry seasons across UDOI filtering levels. UDOI filter indicates level of home range overlap between individuals to count as an edge (e.g., UDOI > 0). Modularity was estimated using walktrap algorithms with 4 and 7 steps to test sensitivity of results to this choice (“4 steps” and “7 steps” in table).*


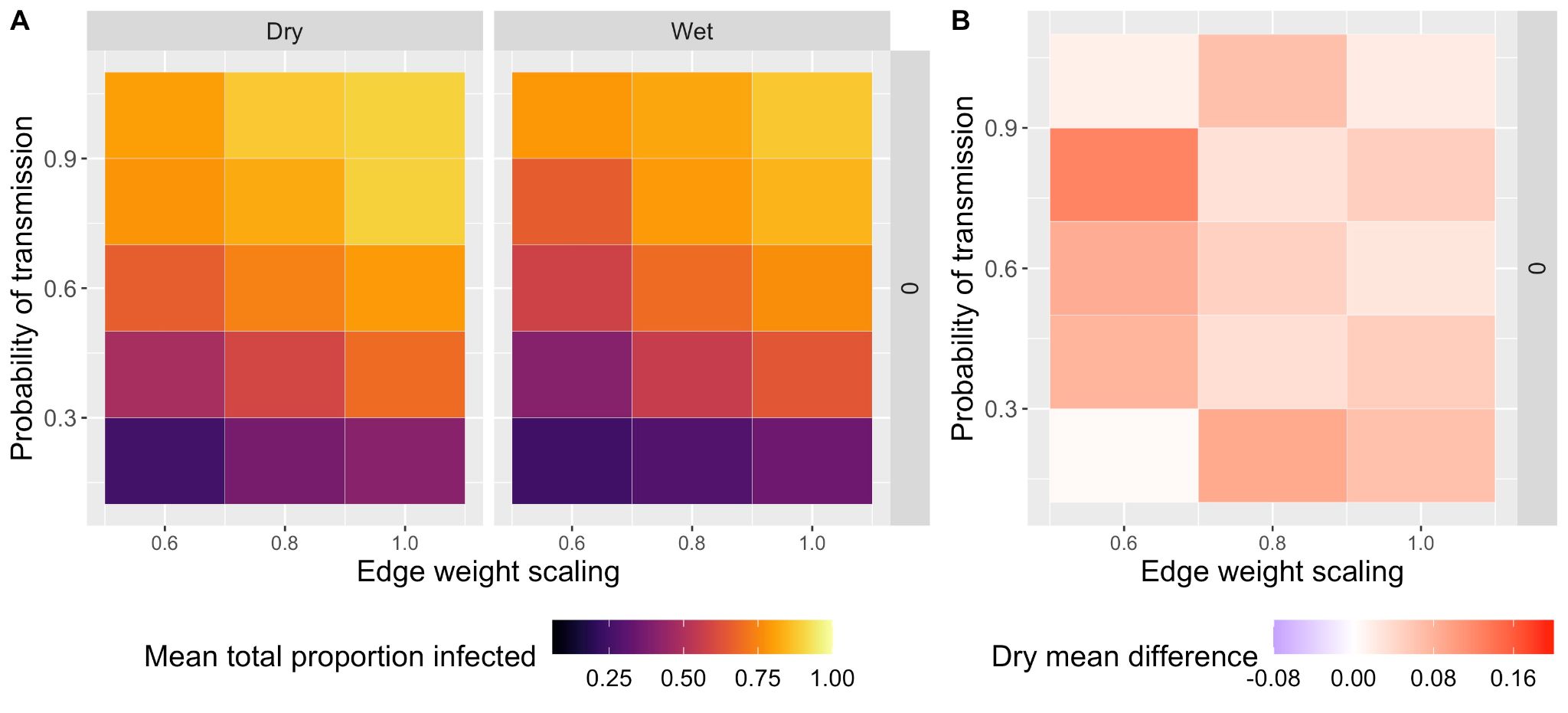


**Figure S17:** Heat maps from the SI model type showing (A) the mean total proportion of individuals infected in a simulated outbreak, and (B) the difference between these mean proportions for dry and wet seasons (relative to the dry season value). The panel row represents the weekly probability of recovery from infection (gamma; equals 0 for SI models in which recovery does not occur). In panel B, red indicates more infections in the dry season, white is no difference, and purple represents more infections in the wet season.

**
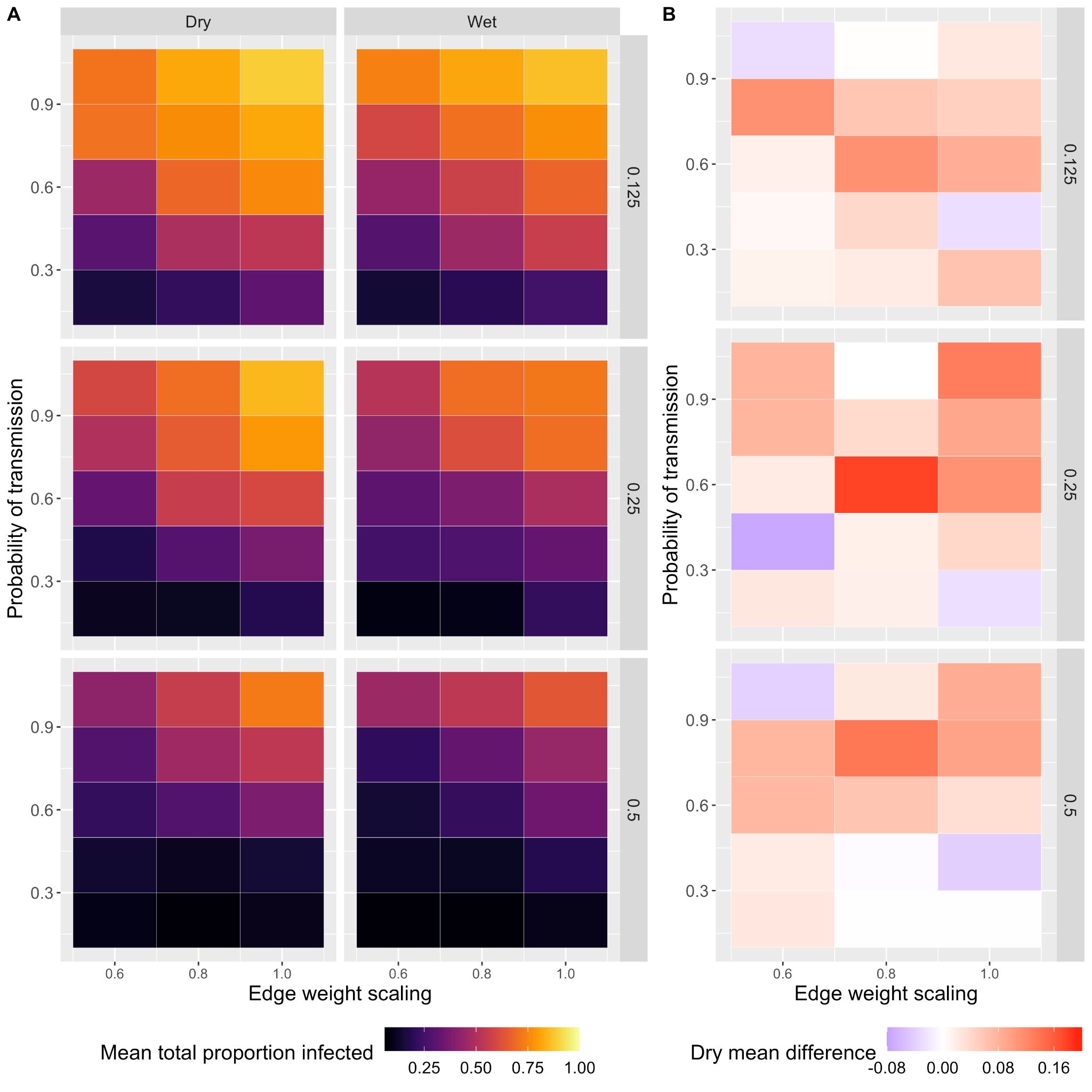
**

**Figure S18:** Heat maps from the SIS model type showing (A) the mean total proportion of individuals infected in a simulated outbreak, and (B) the difference between these mean proportions for dry and wet seasons (relative to the dry season value). Panel rows represent the weekly probability of recovery from infection (gamma). In panel B, red indicates more infections in the dry season, white is no difference, and purple represents more infections in the wet season.


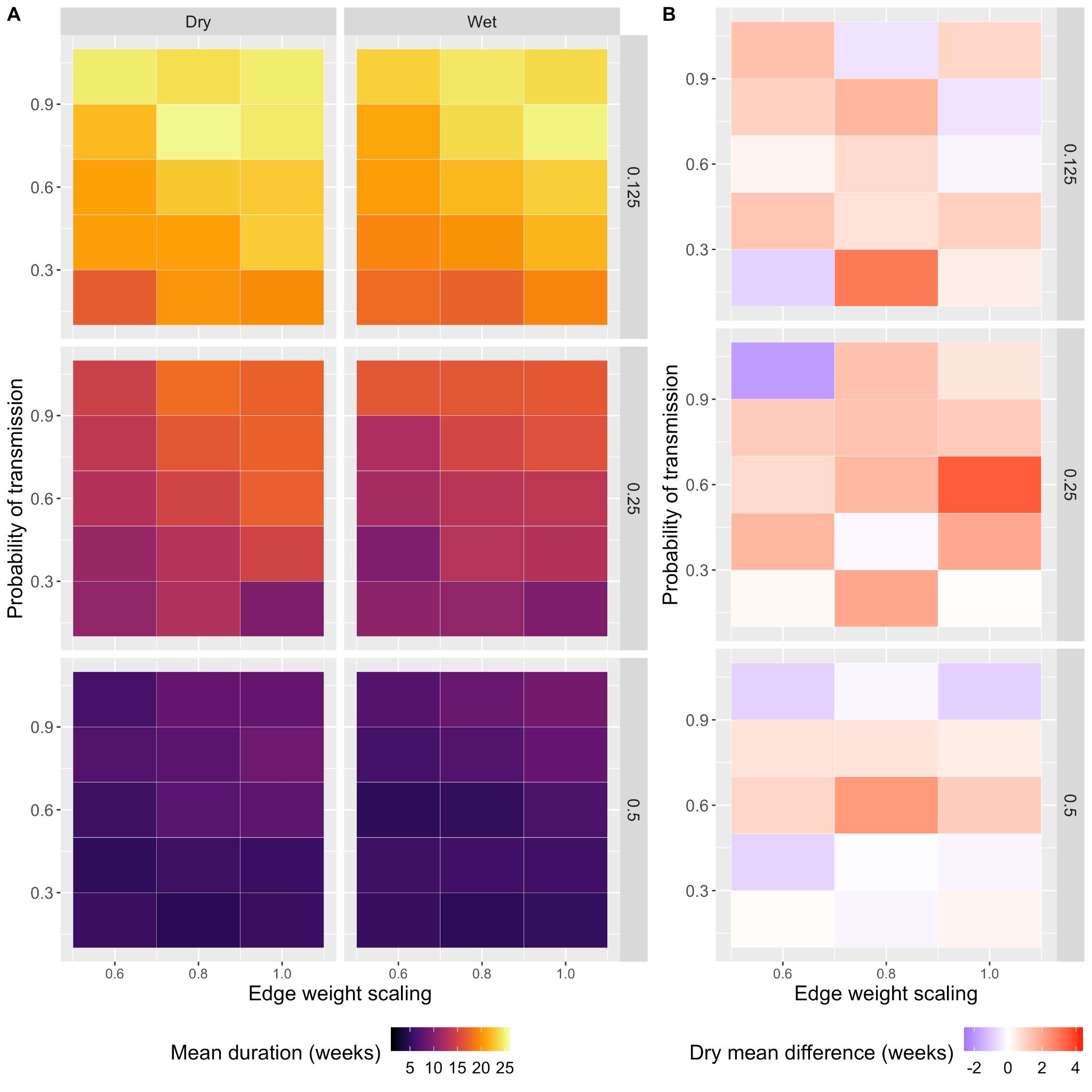


**Figure S19:** Heat maps from the SIR model type showing (A) the mean duration of simulated outbreaks in weeks, and (B) the difference between these means for dry and wet seasons (relative to the dry season value). Panel rows represent the weekly probability of recovery from infection (gamma). In panel B, red indicates longer epidemics in the dry season, white is no difference, and purple represents longer epidemics in the wet season.


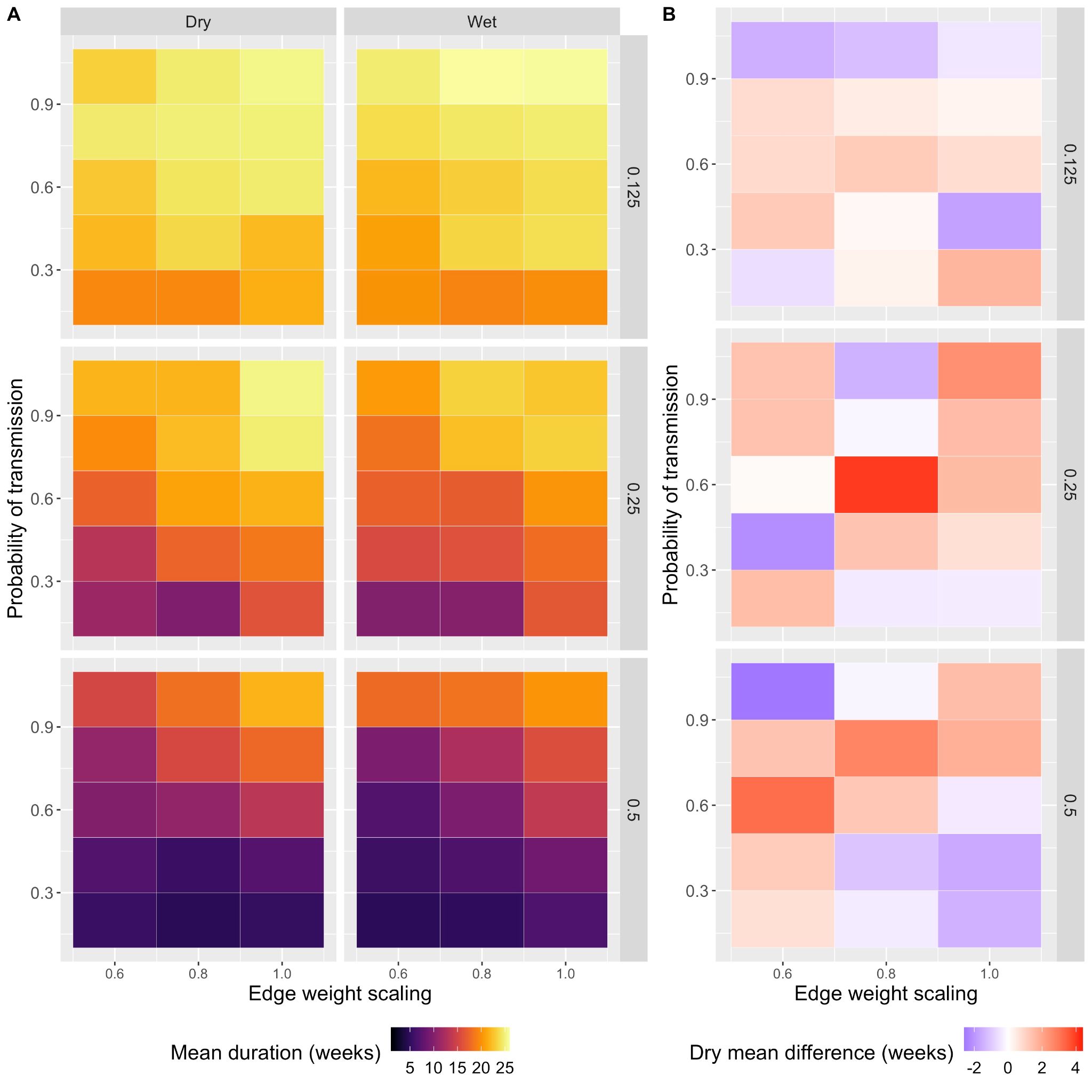


**Figure S20:** Heat maps from the SIS model type showing (A) the mean duration of simulated outbreaks in weeks, and (B) the difference between these means for dry and wet seasons (relative to the dry season value). Panel rows represent the weekly probability of recovery from infection (gamma). In panel B, red indicates longer epidemics in the dry season, white is no difference, and purple represents longer epidemics in the wet season.


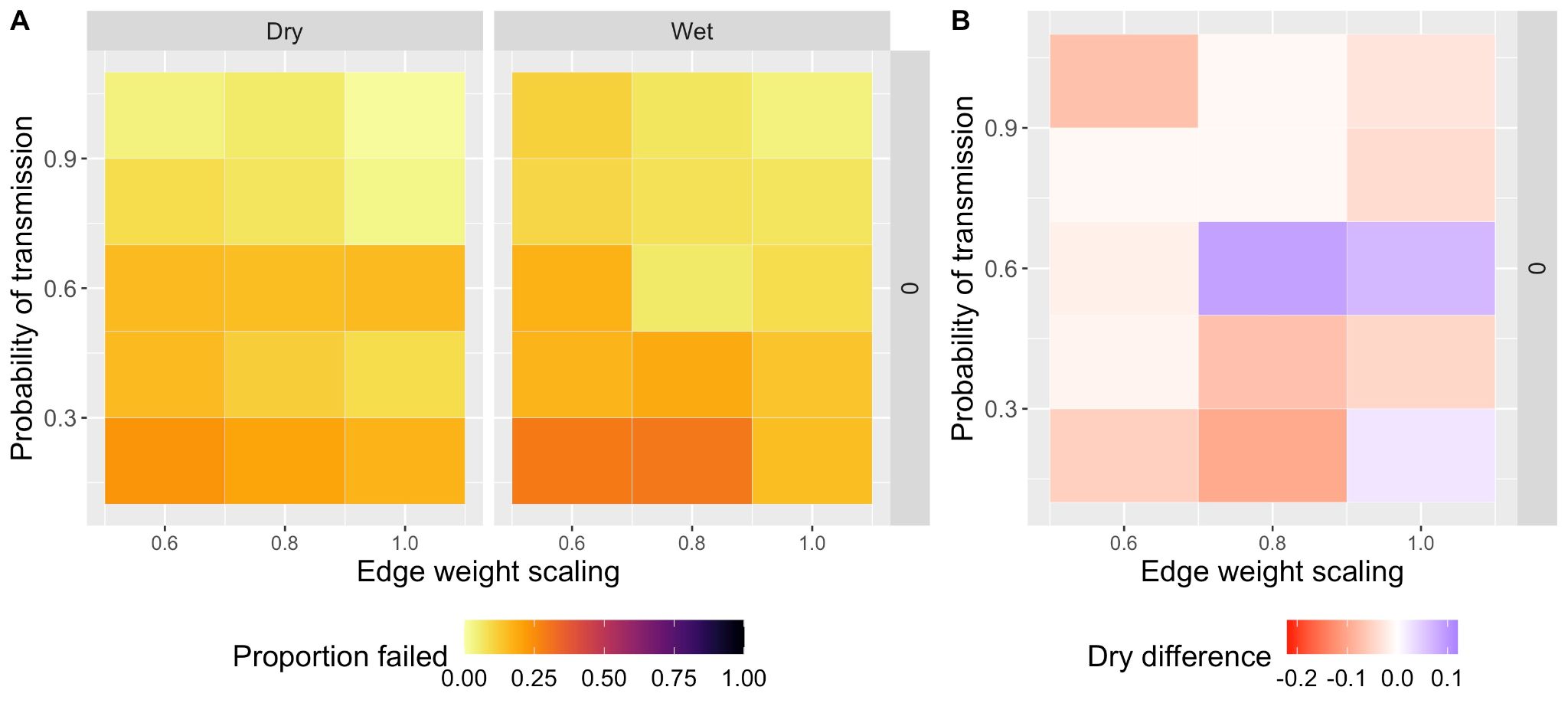


**Figure S21:** Heat maps from the SI model type showing (A) the proportion of failed epidemics (only one individual infected), and (B) the difference between these proportions for dry and wet seasons (relative to the dry season value). The panel row represents the weekly probability of recovery from infection (gamma; equals zero for SI models in which recovery from infection does not occur). In panel B, red indicates a greater proportion of successful epidemics in the dry season, white is no difference, and purple represents a greater proportion of successful epidemics in the wet season.


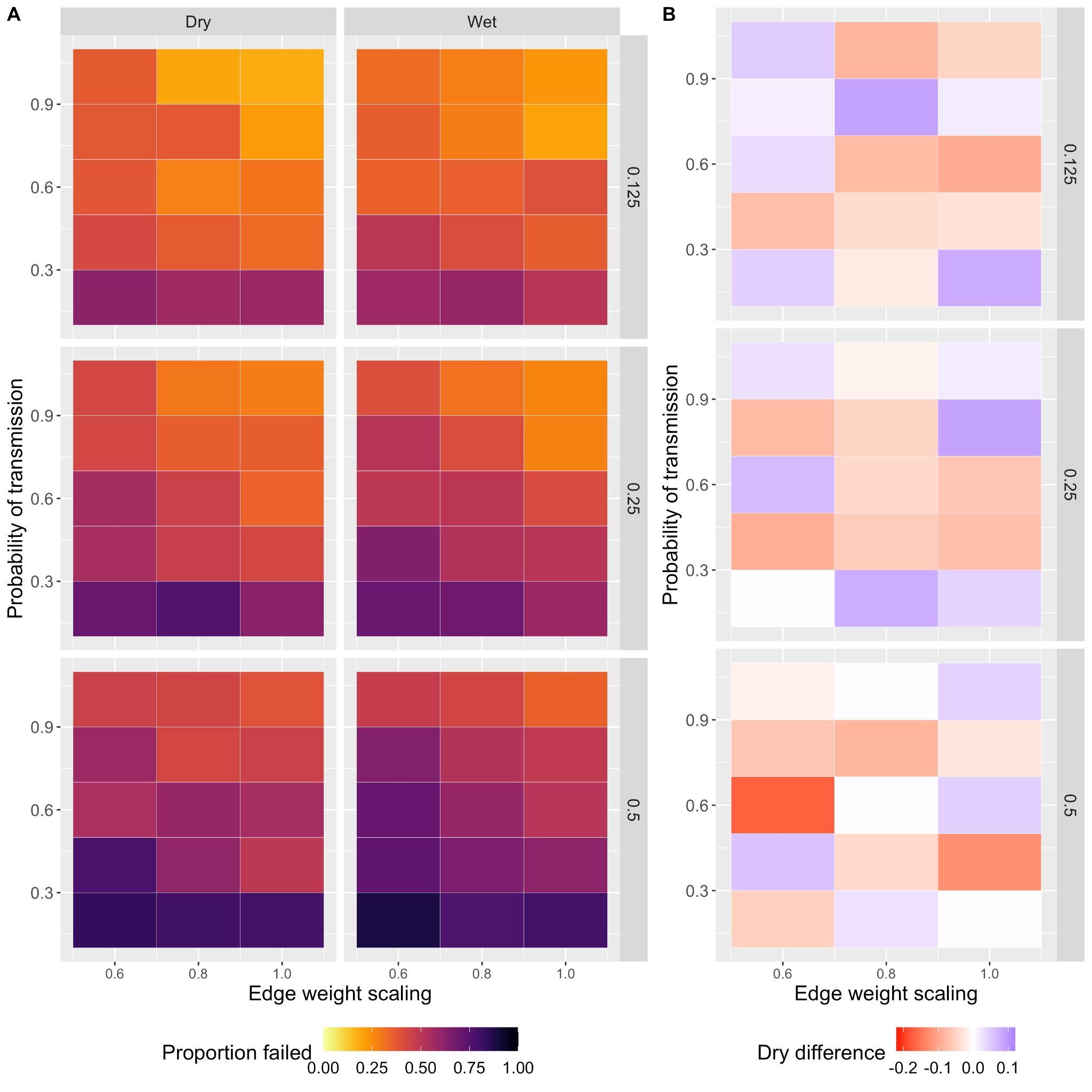


**Figure S22:** Heat maps from the SIR model type showing (A) the proportion of failed epidemics, and (B) the difference between these proportions for dry and wet seasons (relative to the dry season value). Panel rows represent the weekly probability of recovery from infection (gamma). In panel B, red indicates a greater proportion of successful epidemics in the dry season, white is no difference, and purple represents a greater proportion of successful epidemics in the wet season.


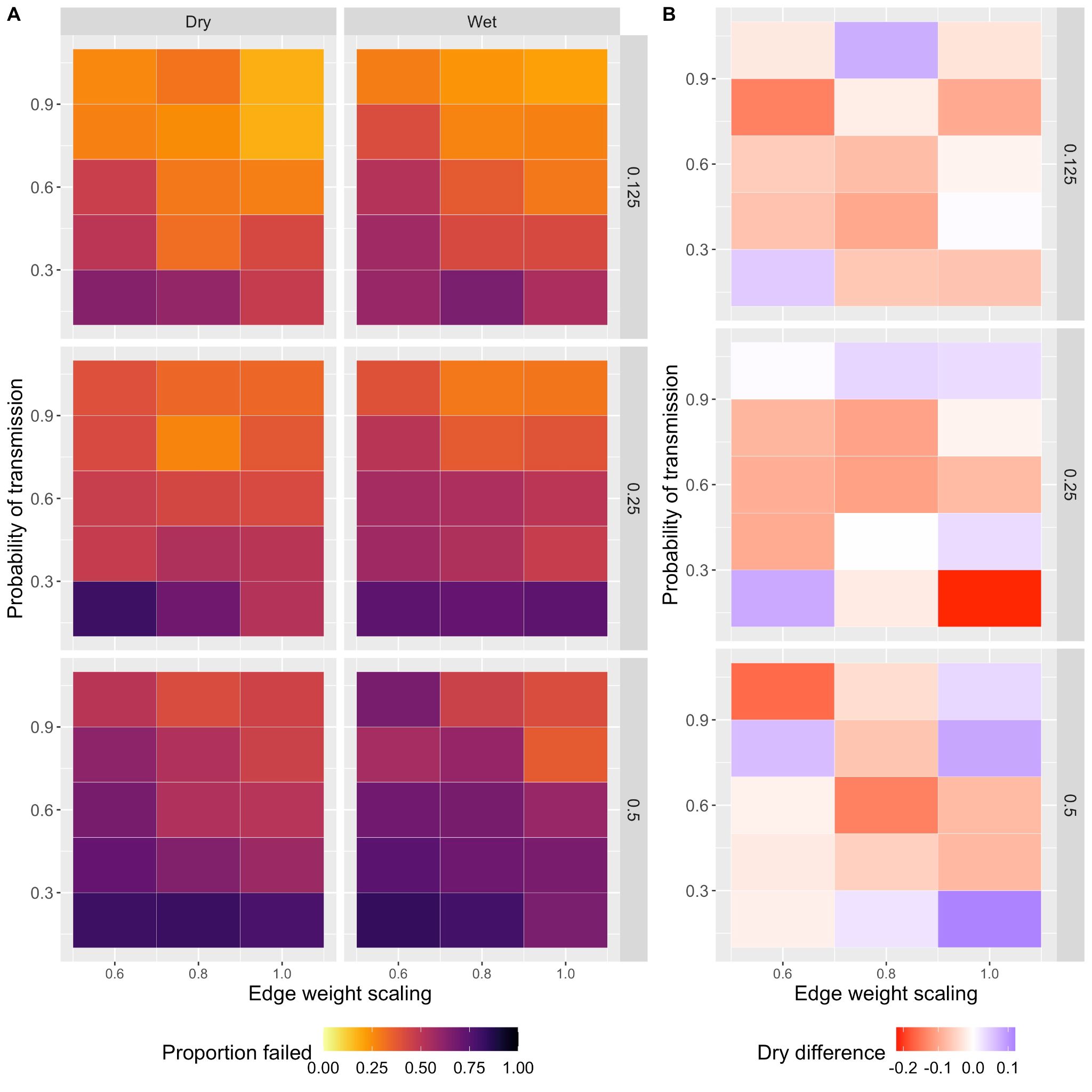


**Figure S23:** Heat maps from the SIS model type showing (A) the proportion of failed epidemics, and (B) the difference between these proportions for dry and wet seasons (relative to the dry season value). Panel rows represent the weekly probability of recovery from infection (gamma). In panel B, red indicates a greater proportion of successful epidemics in the dry season, white is no difference, and purple represents a greater proportion of successful epidemics in the wet season.


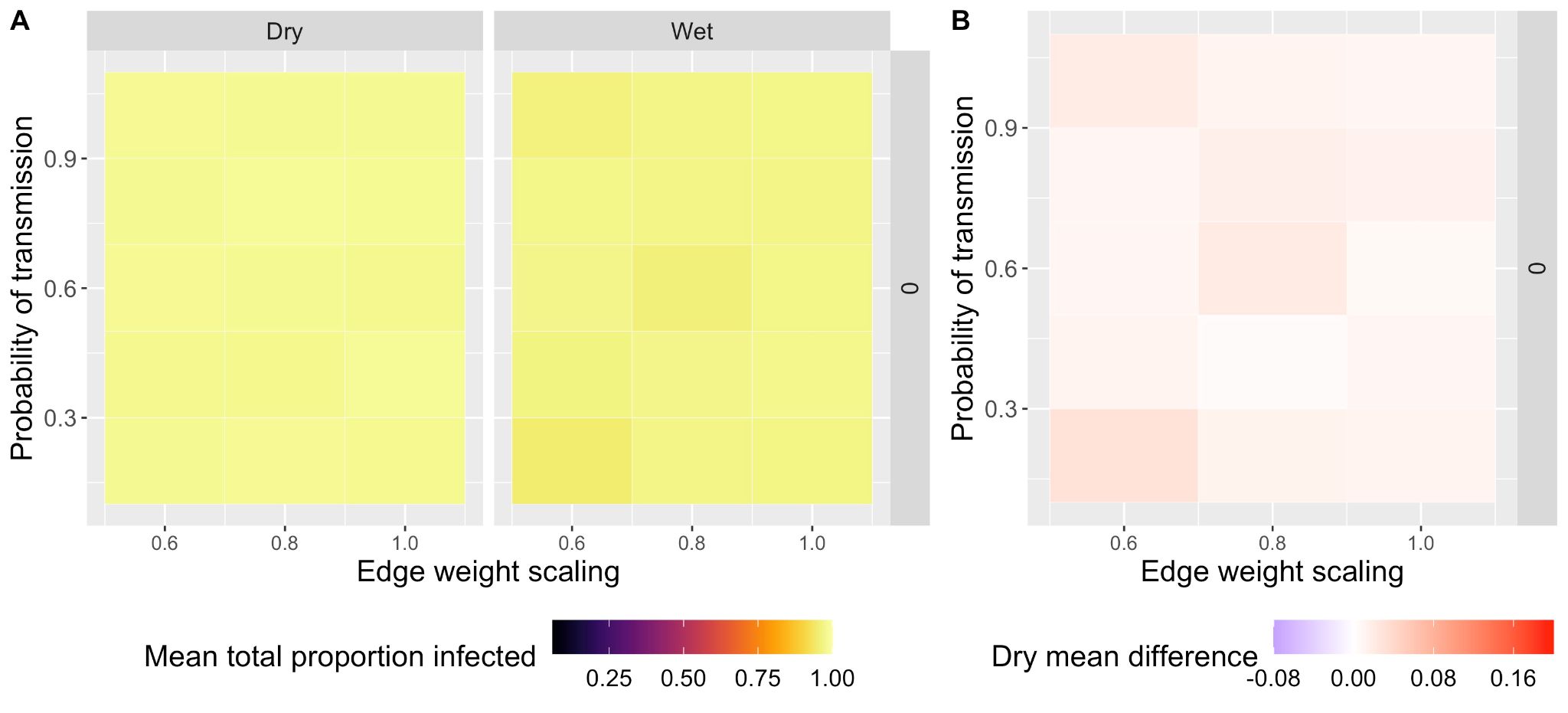


**Figure S24:** Heat maps from the SI model type on binary (unweighted) networks showing (A) the mean total proportion of individuals infected in a simulated outbreak, and (B) the difference between these mean proportions for dry and wet seasons (relative to the dry season value). The panel row represents the weekly probability of recovery from infection (gamma; equals 0 for SI models in which recovery does not occur). In panel B, red indicates more infections in the dry season, white is no difference, and purple represents more infections in the wet season.


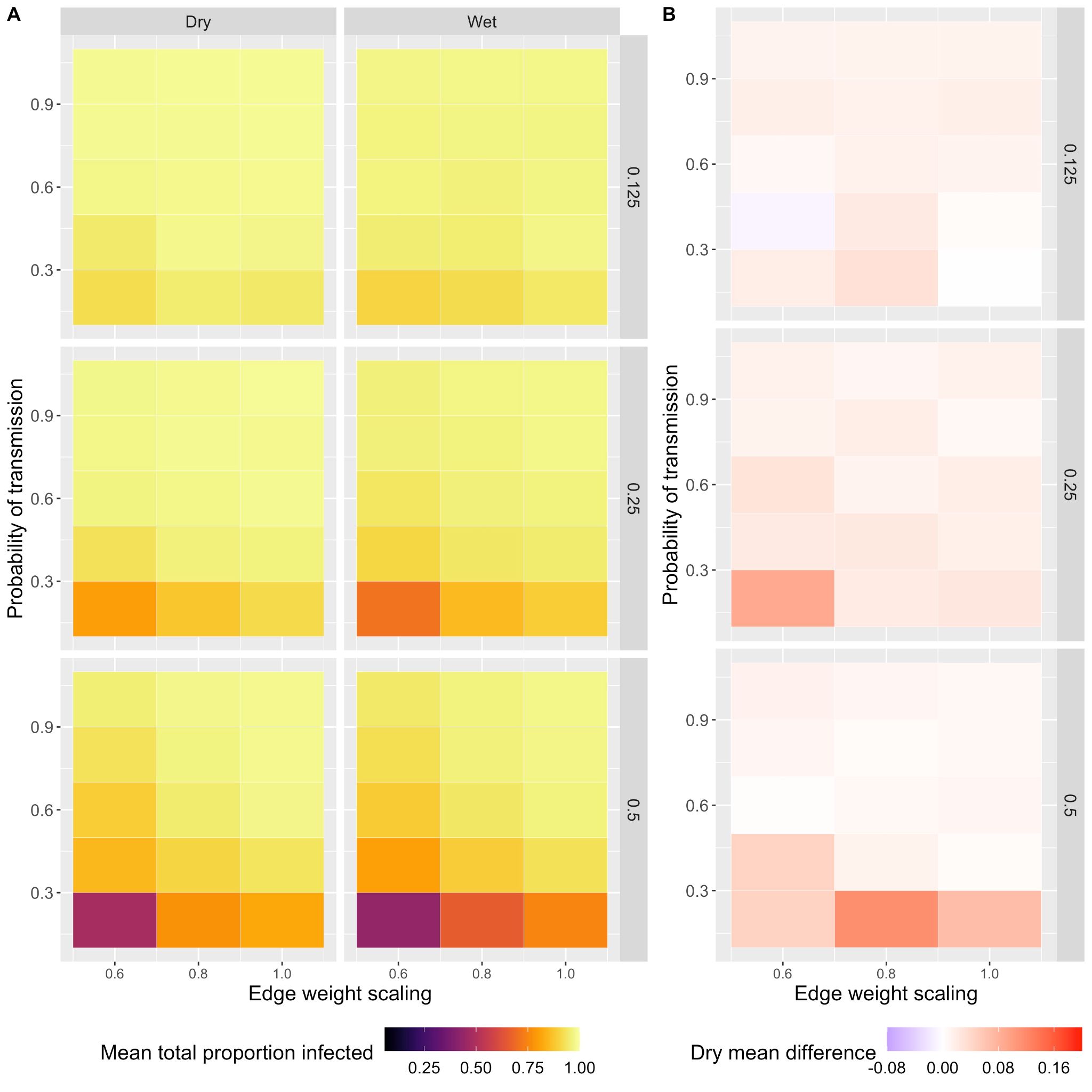


**Figure S25:** Heat maps from the SIR model type on binary (unweighted) networks showing (A) the mean total proportion of individuals infected in a simulated outbreak, and (B) the difference between these mean proportions for dry and wet seasons (relative to the dry season value). Panel rows represent the weekly probability of recovery from infection (gamma). In panel B, red indicates more infections in the dry season, white is no difference, and purple represents more infections in the wet season.


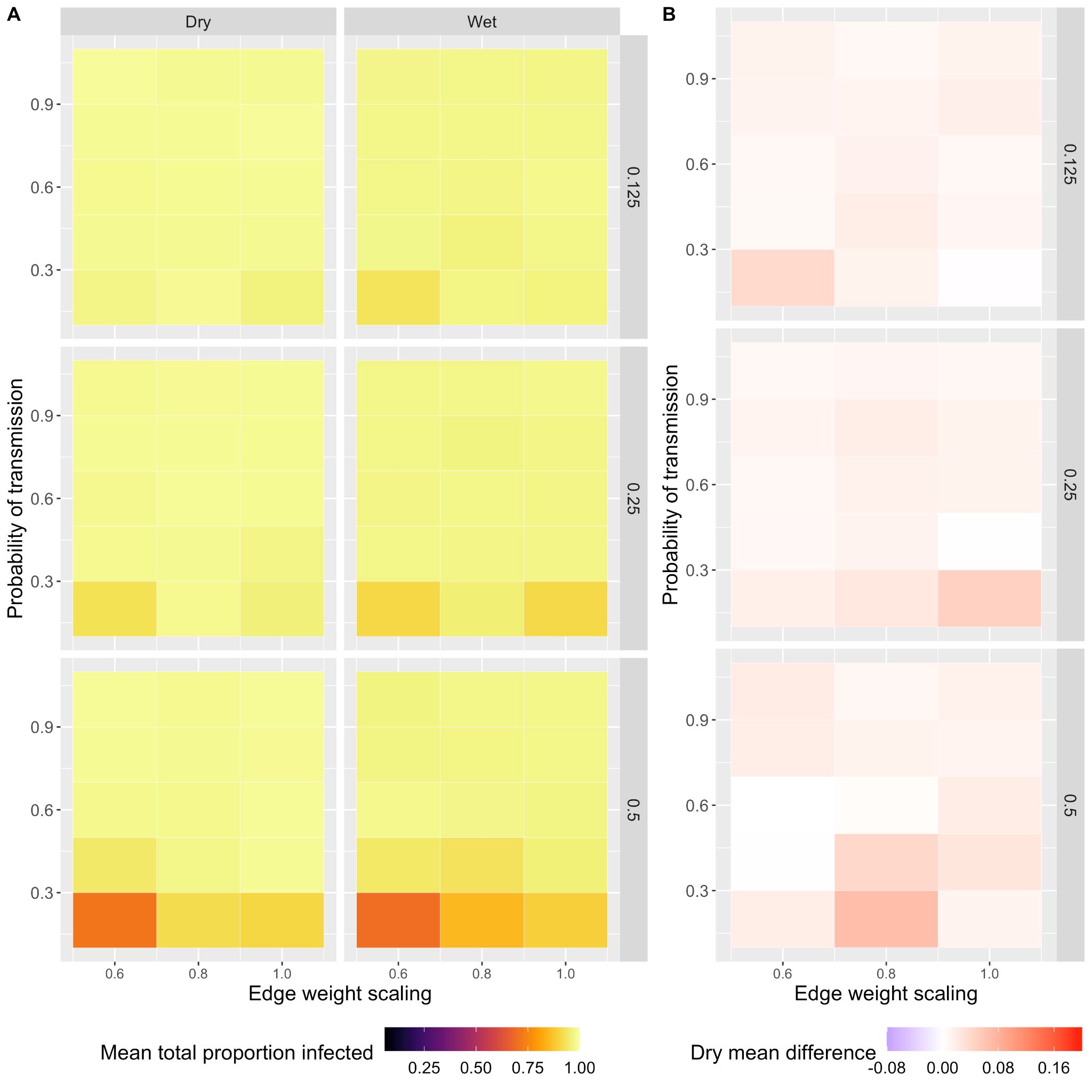


**Figure S26:** Heat maps from the SIS model type on binary (unweighted) networks showing (A) the mean total proportion of individuals infected in a simulated outbreak, and (B) the difference between these mean proportions for dry and wet seasons (relative to the dry season value). Panel rows represent the weekly probability of recovery from infection (gamma). In panel B, red indicates more infections in the dry season, white is no difference, and purple represents more infections in the wet season.


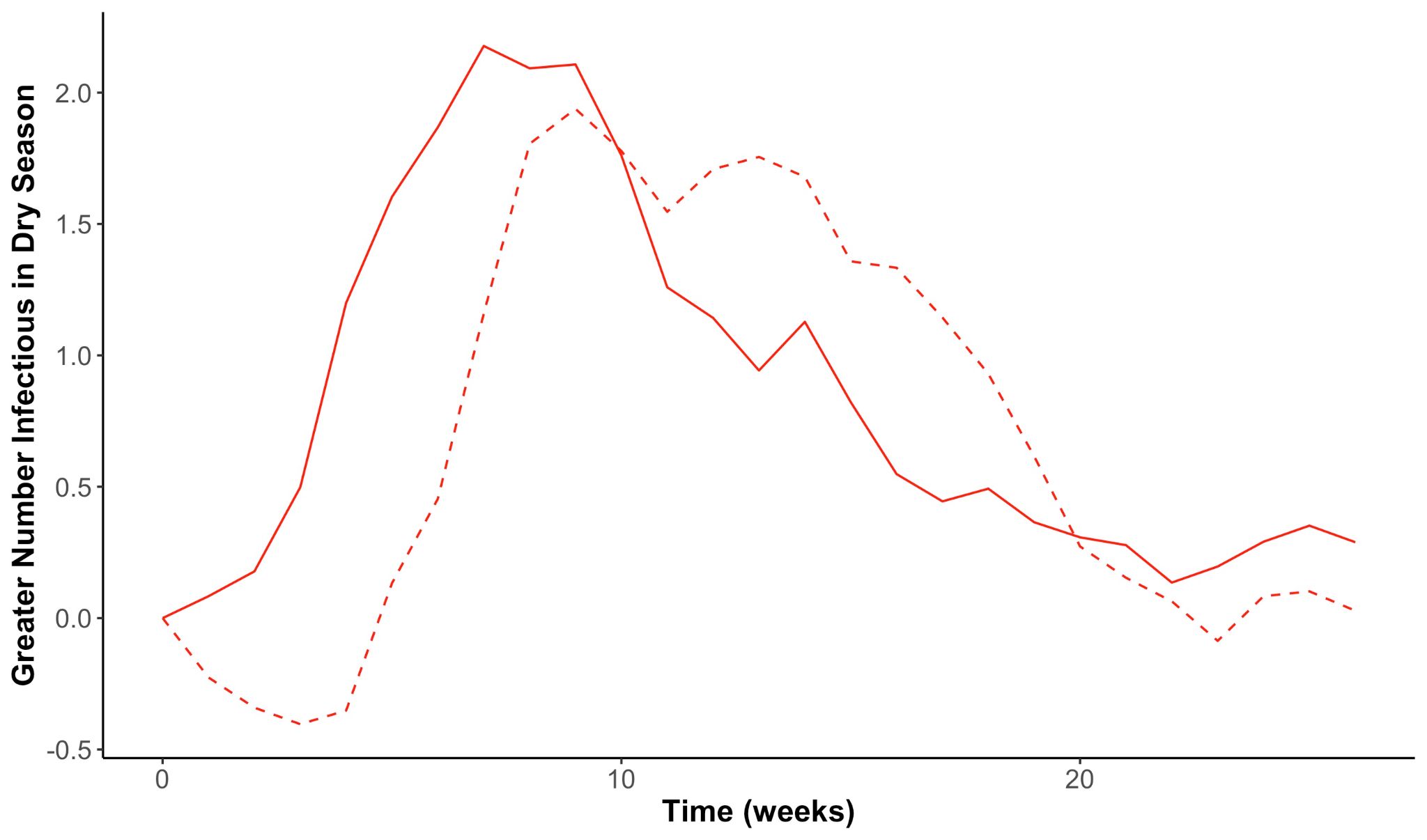


**Figure S27:** Difference between the mean number of infectious individuals in the dry season relative to the wet season over time. The solid line shows these differences for the SIR model scenario with the greatest proportional difference in number of infections (probability of transmission given contact = 0.6, edge weight scaling = 1.0, weekly probability of recovery = 0.25). The dashed line shows the differences for the SIR model scenario that produced the largest epidemics (probability of transmission given contact = 1.0, edge weight scaling = 1.0, weekly probability of recovery = 0.125; see Figure 2 in the main text).


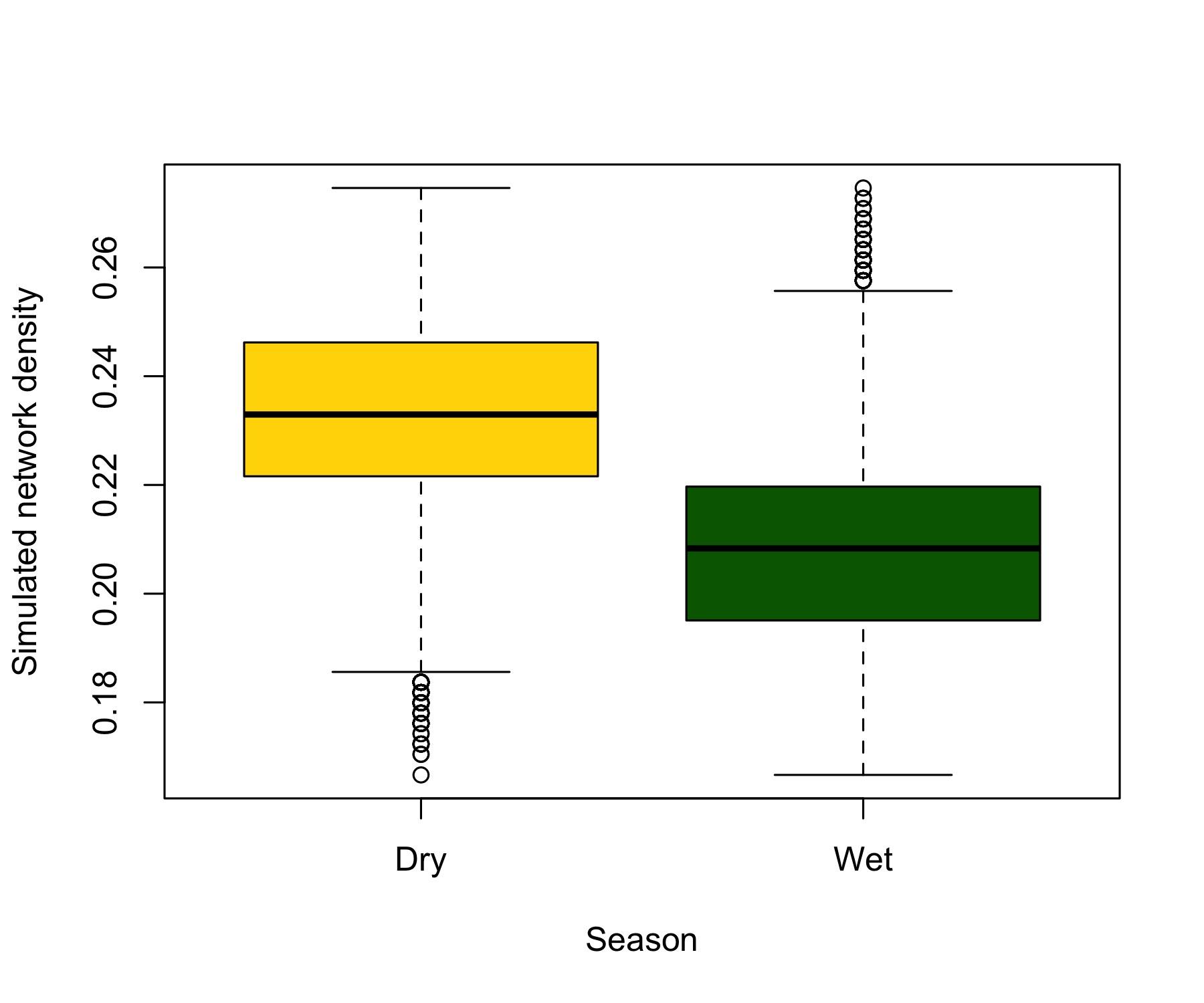


**Figure S28:** Network density of simulated dry and wet season networks. Network density was an emergent property of simulated networks.


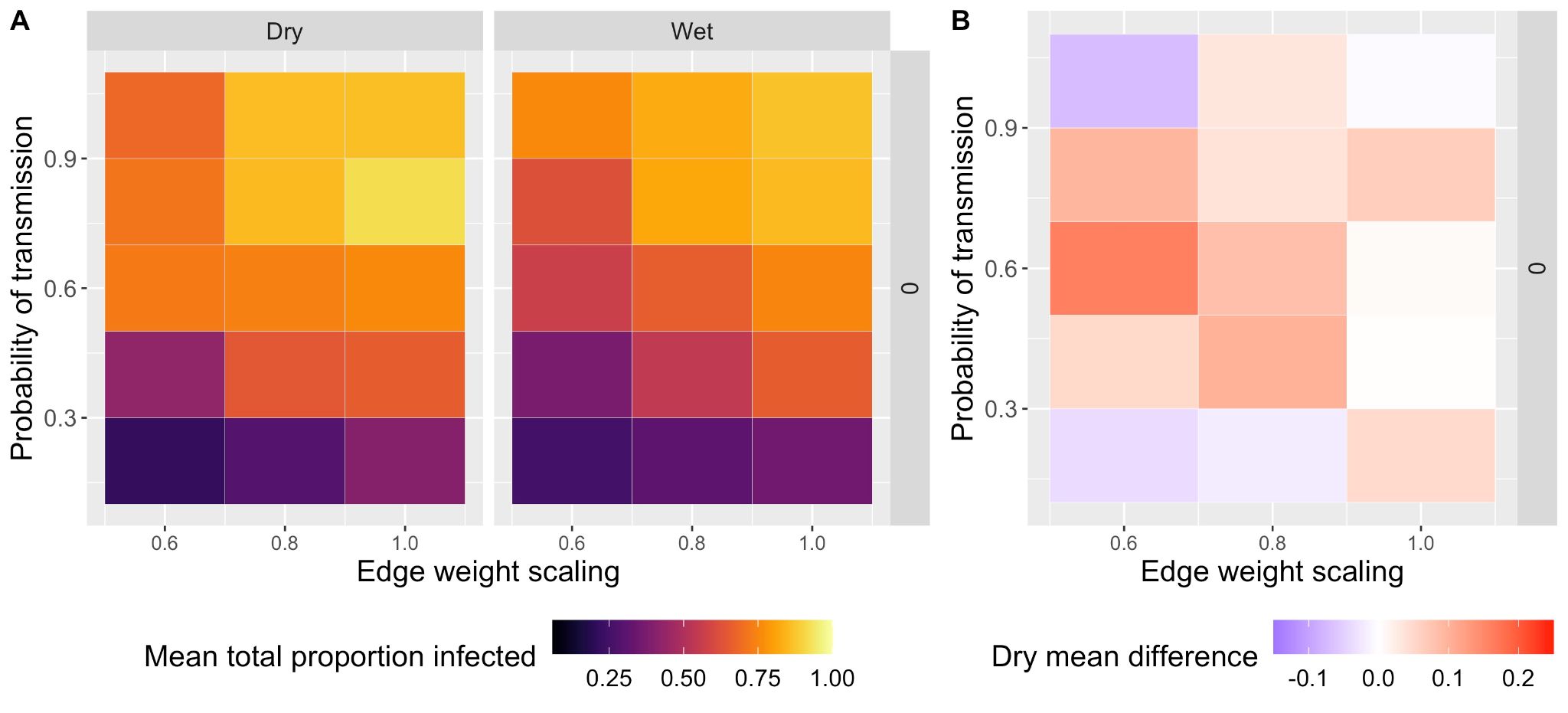


**Figure S29:** Heat maps from the SI model type with only those simulated networks whose modularity value fell within the range of observed modularity values for empirical dry and wet season networks. Heat maps show (A) the mean total proportion of individuals infected in a simulated outbreak, and (B) the difference between these mean proportions for dry and wet seasons (relative to the dry season value). The panel row represents the weekly probability of recovery from infection (gamma; equals 0 for SI models in which recovery does not occur). In panel B, red indicates more infections in the dry season, white is no difference, and purple represents more infections in the wet season.


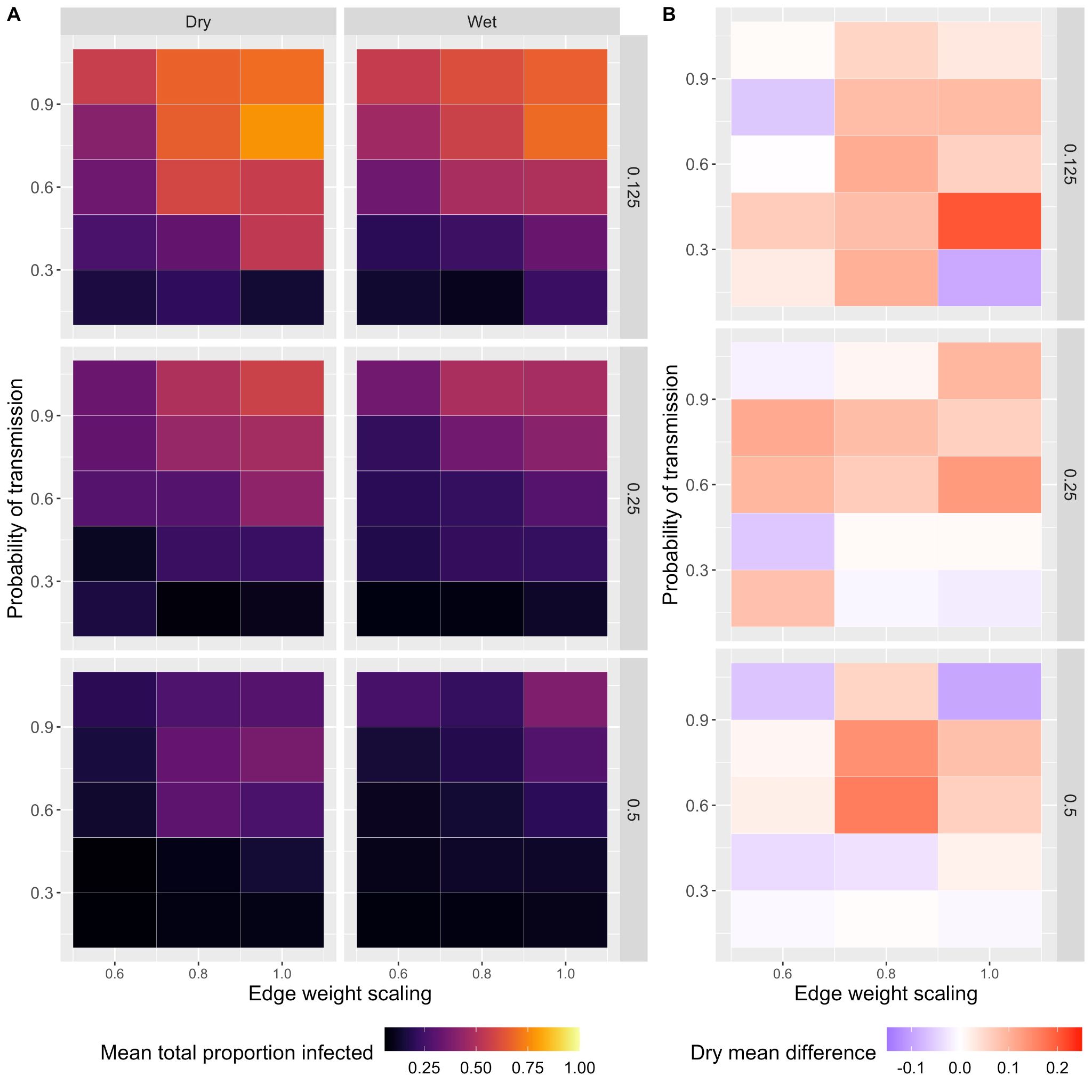


**Figure S30:** Heat maps from the SIR model type with only those simulated networks whose modularity value fell within the range of observed modularity values for empirical dry and wet season networks. Heat maps show (A) the mean total proportion of individuals infected in a simulated outbreak, and (B) the difference between these mean proportions for dry and wet seasons (relative to the dry season value). Panel rows represent the weekly probability of recovery from infection (gamma). In panel B, red indicates more infections in the dry season, white is no difference, and purple represents more infections in the wet season.


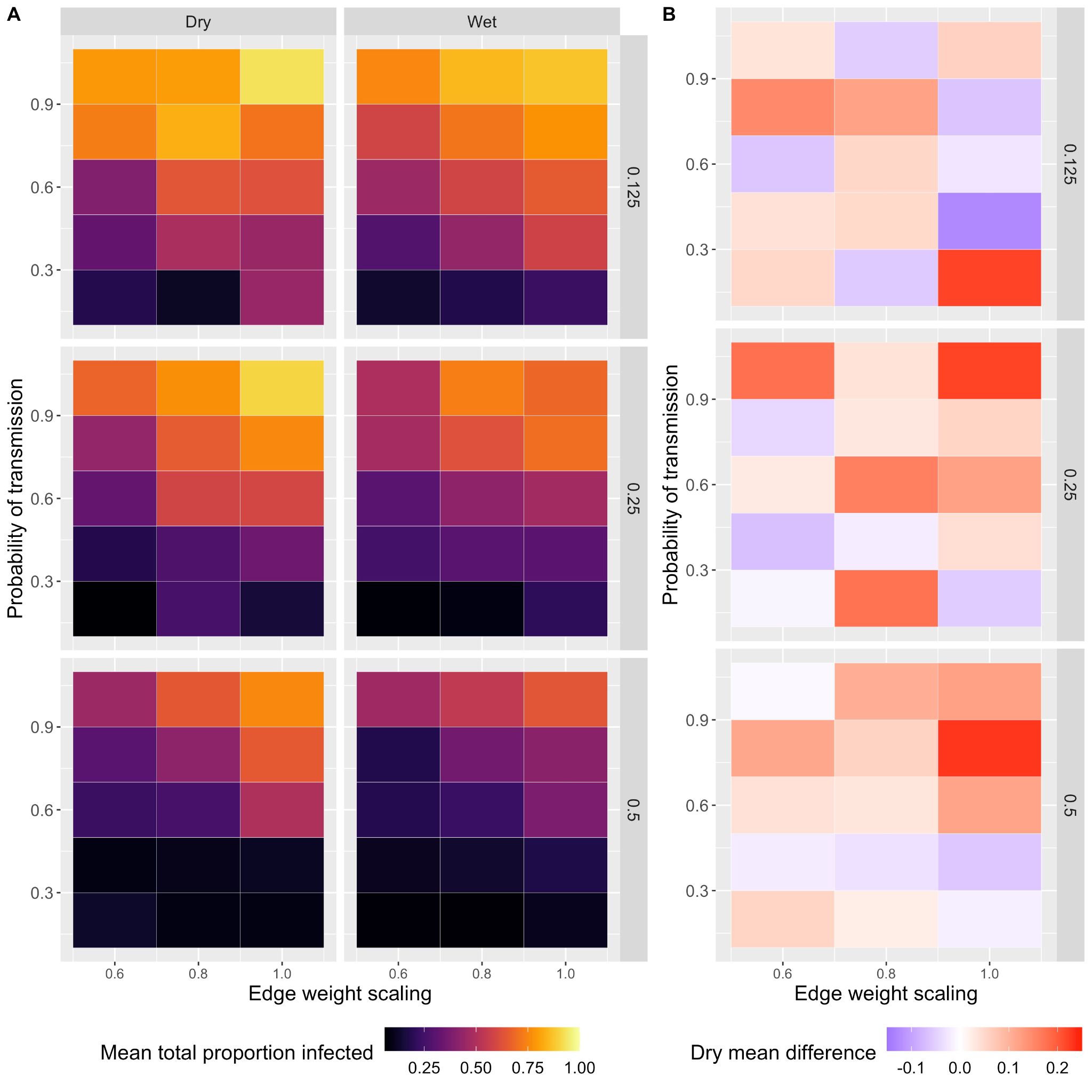


**Figure S31:** Heat maps from the SIS model type with only those simulated networks whose modularity value fell within the range of observed modularity values for empirical dry and wet season networks. Heat maps show (A) the mean total proportion of individuals infected in a simulated outbreak, and (B) the difference between these mean proportions for dry and wet seasons (relative to the dry season value). Panel rows represent the weekly probability of recovery from infection (gamma). In panel B, red indicates more infections in the dry season, white is no difference, and purple represents more infections in the wet season.


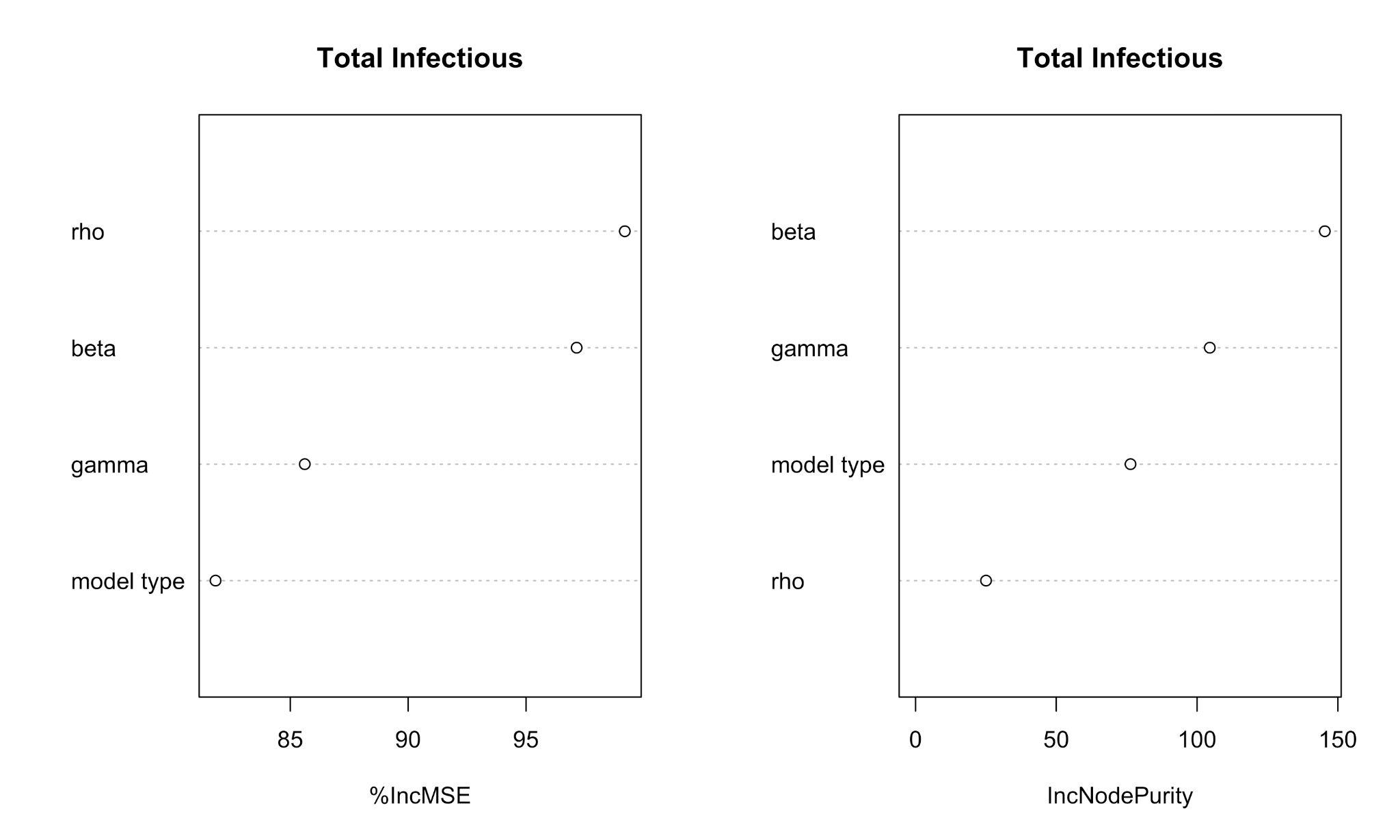


**Figure S32:** Variable importance plots for the random forest regression for the total proportion of the population ever infectious. Parameter names are given on the y-axis (see Table 1 in the main text for parameter definitions). Mean decrease in accuracy is given on the x-axis in the left panel; mean decrease in node impurity is given on the x-axis in the right panel.


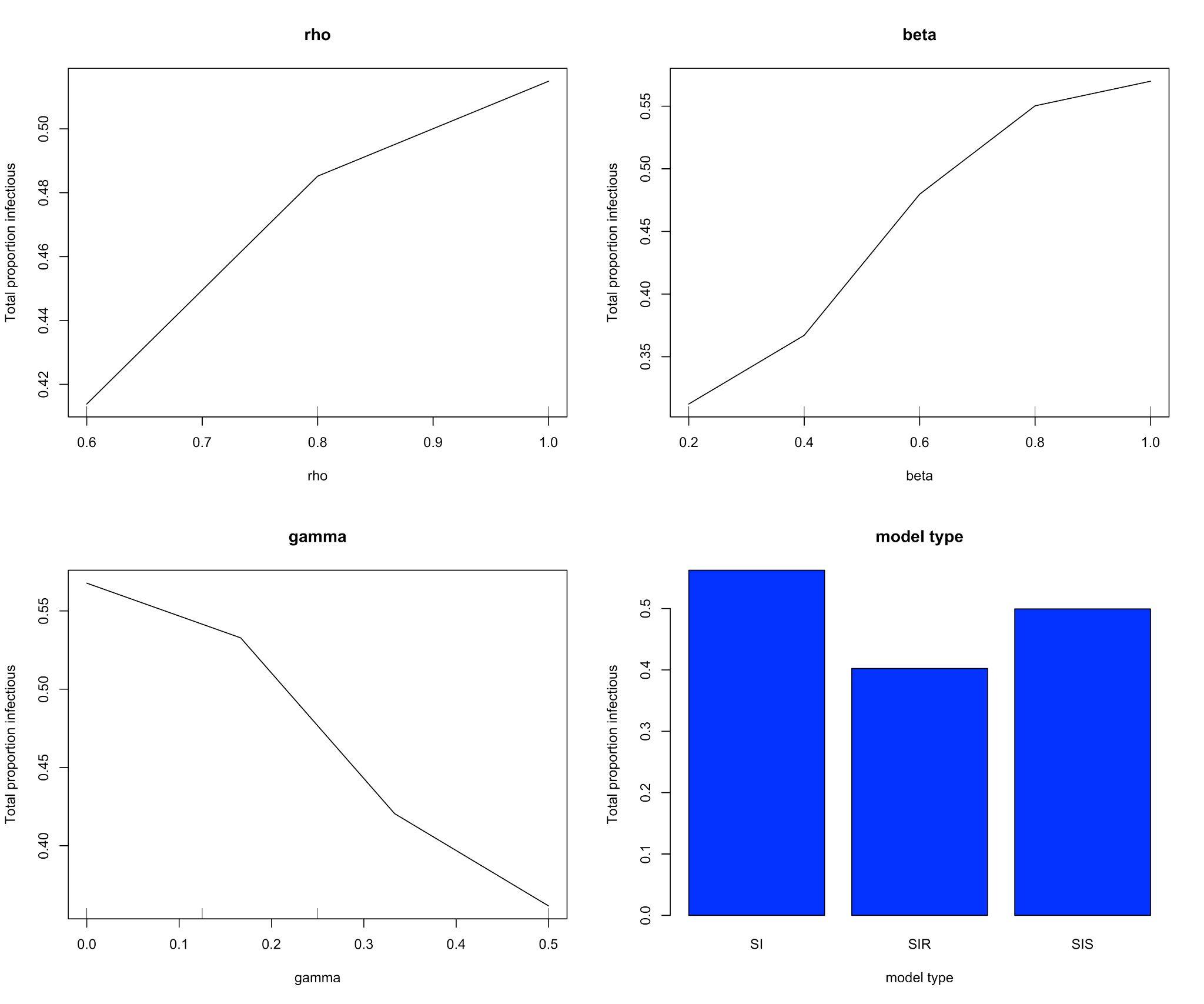


**Figure S33:** Partial importance plots for the random forest regression for the total proportion of the population ever infectious. Parameter names are given in the titles and x-axes (see Table 1 in the main text for parameter definitions). Parameters are ordered (top left to right to bottom left to right) based on variable importance, as determined by mean decrease in accuracy (see Figure S16, left panel).


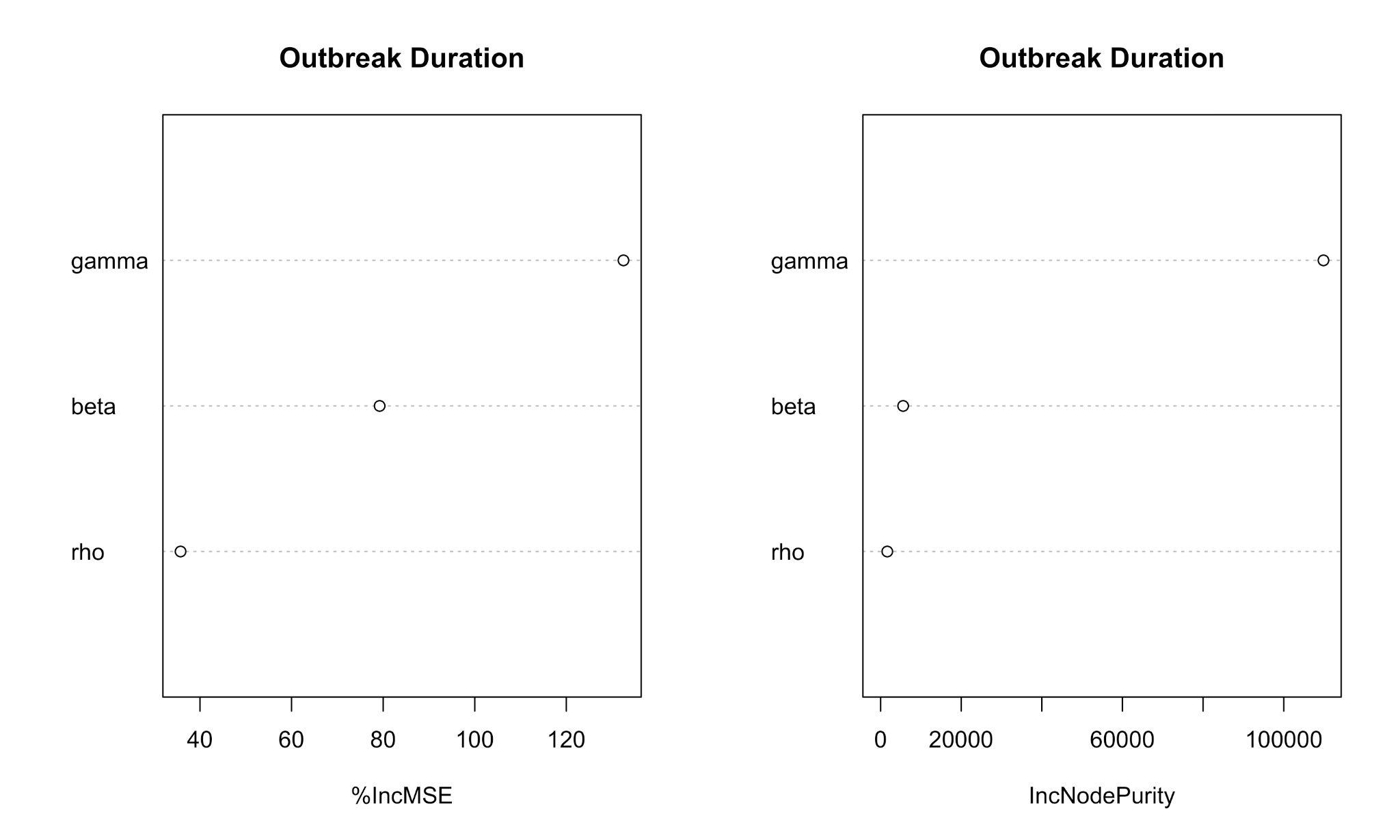


**Figure S34:** Variable importance plots for the random forest regression for the duration of simulated SIR outbreaks. Parameter names are given on the y-axis (see Table 1 in the main text for parameter definitions). Mean decrease in accuracy is given on the x-axis in the left panel; mean decrease in node impurity is given on the x-axis in the right panel.


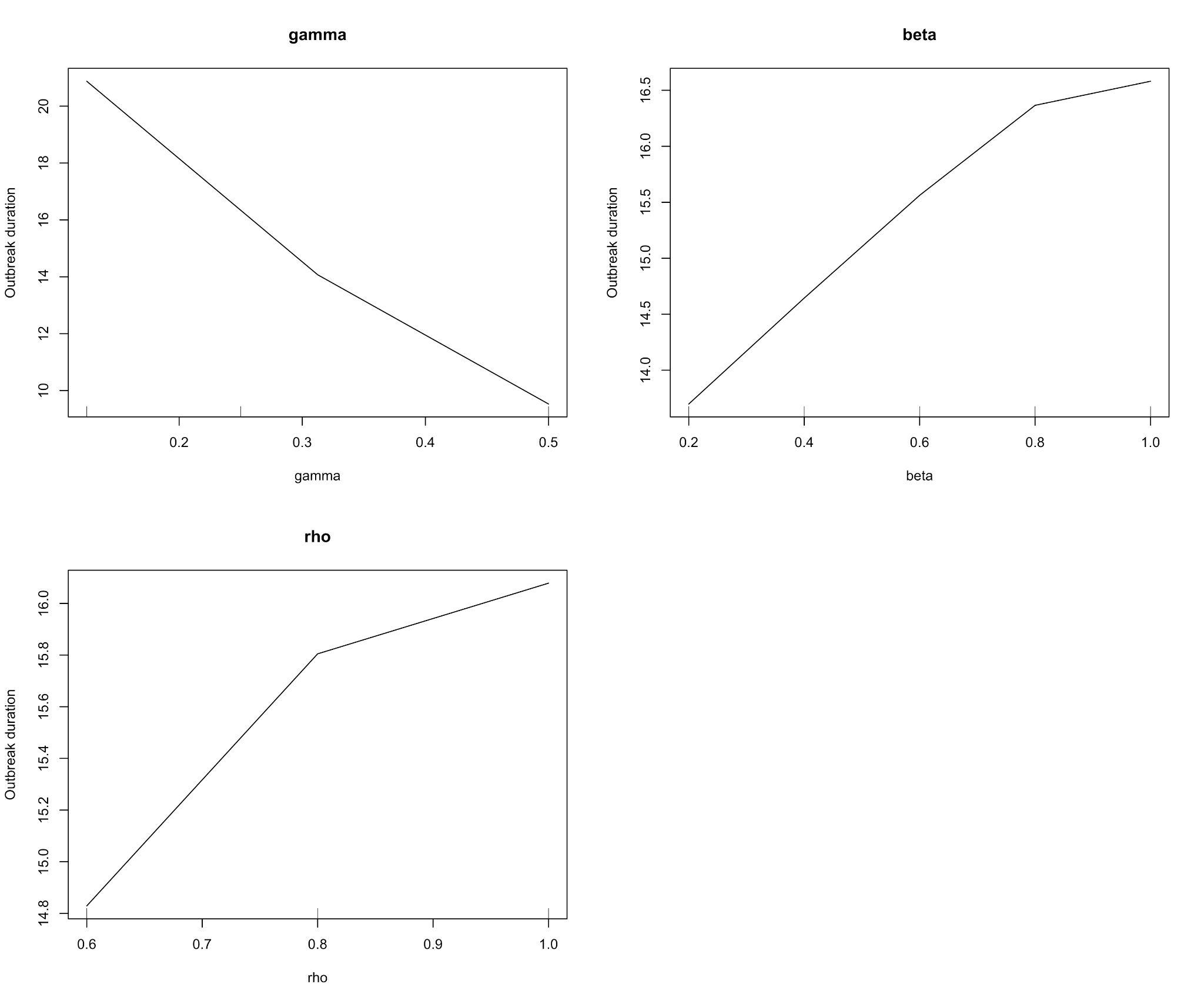


**Figure S35:** Partial importance plots for the random forest regression for the duration of simulated SIR outbreaks. Parameter names are given in the titles and x-axes (see Table 1 in the main text for parameter definitions). Parameters are ordered (top left to right to bottom left to right) based on variable importance, as determined by mean decrease in accuracy (see Figure S18, left panel).
